# Prmt6 Deficiency Drives Osteoblast Senescence Promoting Age‐Related Osteoporosis via Epigenetic Remodelling of the H3R2me2a/Sting/Ifitm3 Pathway

**DOI:** 10.1111/jcmm.71308

**Published:** 2026-09-18

**Authors:** Yuxue Wang, Xiaoyong Ge, Shengjie Chang, Diankai Wang, Ke Xu, Haowei Xu, Xiaowei Liu, Shanjin Wang

**Affiliations:** ^1^ Shanghai East Hospital Postgraduate Training Base Jinzhou Medical University Shanghai China; ^2^ Department of Osteoporosis and Bone Diseases PudongNew Area Geriatric Hospital Shanghai China; ^3^ Department of Spinal Surgery, Shanghai East Hospital, School of Medicine Tongji University Shanghai China

**Keywords:** H3R2me2a, Ifitm3, osteoblasts, osteoporosis, Prmt6, Sting

## Abstract

Protein arginine methyltransferase 6 (Prmt6), an epigenetic regulator, plays an unclear role in bone homeostasis and osteoblast senescence. In this study, we employed Western blot, qPCR, immunofluorescence, SA‐β‐Gal staining, and RNA‐seq to systematically investigate the regulatory mechanism of the Prmt6/H3R2me2a/Sting/Ifitm3 axis in osteoblast senescence, and further validated the association of relevant molecules with osteoporosis at the animal level using immunohistochemistry and micro‐CT. Our results showed that Prmt6 deficiency led to bone loss and induced osteoblast senescence in mice. Mechanistically, Prmt6 ablation was accompanied by reduced H3R2me2a modification and increased chromatin accessibility at the Sting promoter, suggesting that this histone mark may restrict local chromatin opening, thereby triggering sustained activation of the Sting pathway and marked upregulation of its classical downstream target gene Ifitm3. Functional experiments confirmed that inhibiting Sting or knocking down Ifitm3 effectively alleviated the senescent phenotype of osteoblasts. Consistently, in aged bone tissues, Prmt6 and H3R2me2a expression were decreased, while Sting and Ifitm3 levels were elevated. Collectively, this study reveals a previously unreported Prmt6/H3R2me2a/Sting/Ifitm3 signalling axis that maintains osteoblast homeostasis by constraining excessive activation of the Sting/Ifitm3 axis via epigenetic repressive modifications, offering a novel target for epigenetic intervention in osteoporosis.

## 1 Introduction

Osteoporosis is a degenerative bone disease closely associated with ageing [1], characterized by reduced bone mineral density and deteriorated bone microarchitecture [2], leading to increased bone fragility [3] and a significantly elevated risk of fractures [4]. Osteoporosis has become a significant health concern among individuals aged 50 and above [5, 6], with middle‐aged and elderly women being particularly affected [7, 8]. The prevalence of osteoporosis among individuals aged 65 and older reaches 32.0%, with 10.7% among men and 51.6% among women [9]. As global population ageing intensifies, osteoporosis has become an increasingly severe public health issue.

Within the skeletal system, maintaining bone homeostasis relies on a delicate balance between osteoblast‐mediated bone formation and osteoclast‐mediated bone resorption [10, 11]. Osteoporosis in the elderly is a classic disease resulting from the disruption of this balance, characterized by an imbalance between bone formation and bone resorption [12]. This imbalance leads to a significant reduction in bone mass, deterioration of bone microarchitecture, and increased bone fragility [13]. Research in this field frequently employs preclinical models based on cellular senescence to investigate underlying mechanisms. These studies have revealed multiple core pathological mechanisms, including the accumulation of senescent cells, osteoblast‐to‐adipocyte lineage conversion, suppression of Wnt signalling, and increased osteoclastogenesis [14]. In this complex series of ageing processes, osteoblast senescence has been identified as a key factor driving age‐related reductions in bone formation [15] and disruptions in bone homeostasis [16]. Ageing osteoblasts not only exhibit diminished proliferation and differentiation capabilities and impaired bone matrix synthesis, but also secrete various inflammatory cytokines and chemokines. Therefore, osteoblast senescence in osteoporosis warrants further investigation.

The occurrence of cellular senescence is closely associated with multiple intrinsic molecular mechanisms, including telomere shortening, accumulation of DNA damage, genomic instability, mitochondrial dysfunction, and epigenetic alterations [17]. Epigenetic regulation plays a key role in the ageing process [18]. Research indicates that global chromatin restructuring is prevalent in senescent cells [19], including alterations in histone modifications, loss of heterochromatin, and changes in chromatin accessibility [20]. Asymmetric dimethylation of arginine 2 on histone H3 (H3R2me2a) is catalysed by the protein arginine methyltransferase (PRMT) family [21] and participates in regulating various biological processes, including gene transcription, DNA damage repair, and cell fate determination [22]. Among various epigenetic modifications, H3R2me2a has been studied in inhibiting hepatocellular carcinoma tumour growth [23] and inducing apoptosis in endometrial carcinoma cells through histone modifications [24]. However, its specific function and regulatory mechanisms in osteoblast senescence remain an under‐explored field. Protein arginine methyltransferase 6 (Prmt6) is the sole enzyme catalysing the formation of H3R2me2a [22, 25]. The known functions of Prmt6 in tumorigenesis, cell cycle regulation, and other areas have provided new research perspectives [26]. In cancer and other systems, Prmt6 has been demonstrated to be a key negative regulator of cellular senescence. Through its epigenetic activities—such as depositing H3R2me2a—it suppresses the expression of senescence‐related genes like P53 and P21, thereby sustaining cell proliferation and resisting senescence [22, 27]. In the field of neurobiology, research on Prmt6 has revealed its multifaceted functions: it participates in neurogenesis and plays a crucial regulatory role in neuropathic pain; notably, within dorsal root ganglia, Prmt6 regulates the expression of heterochromatin nuclear ribonucleoprotein F (hnRNP F) through a unique mechanism independent of its classical H3R2 methylation activity (dependent on its amino acid domain 319–388) [28], thereby influencing pain signalling. However, this crucial Prmt6‐mediated H3R2me2a anti‐ageing epigenetic mechanism has not yet been investigated in the field of bone biology, particularly regarding its potential regulatory role in osteoblast senescence and age‐related osteoporosis.

Sting (stimulator of interferon genes), as the core component of the cGAS‐STING pathway, has seen its research rapidly expand beyond anti‐infective immunity into multiple fields including oncology, autoimmune diseases, ageing, and bone metabolism [29]. Sting is a core adaptor protein in the innate immune pathway that typically senses cGAMP in the cytoplasm and triggers the type I interferon response [30]. In non‐osteoporosis settings, Sting serves as both a key driver of inflammation and autoimmune diseases (such as systemic lupus erythematosus) [31] and a critical target for tumour immunotherapy, with its agonists (e.g., GNE‐6468) attracting significant development interest [32, 33]. Concurrently, activation of the non‐canonical cGAS‐STING pathway has recently been recognized as a pivotal mechanism driving ageing and associated inflammatory phenotypes [34]. Unlike the classical pathogen response, Sting activation in the context of ageing exhibits non‐canonical characteristics: it may not depend on the classical cGAMP‐STING nuclear localization pathway, but rather correlates with abnormal localization in the endoplasmic reticulum and chromatin, persistently driving low‐grade inflammation and SASP expression [35]. Interferon‐induced transmembrane protein 3 (Ifitm3) serves as a crucial immune effector molecule involved in viral defence, cell adhesion, membrane fusion, and cholesterol metabolism. Recent studies indicate that Ifitm3 is similarly upregulated in cellular senescence and age‐related diseases such as vascular calcification. It may accelerate cellular senescence by altering cell membrane fluidity, promoting lysosomal activity, and disrupting autophagy flux [36, 37]. However, whether Prmt6 deficiency regulates Sting's chromatin accessibility and transcription through H3R2me2a‐mediated epigenetic mechanisms, thereby inducing Ifitm3 expression and ultimately triggering osteoblast senescence, remains to be systematically elucidated.

This study aims to elucidate a novel molecular regulatory axis involving Prmt6/H3R2me2a/Sting/Ifitm3. This axis relies on the disrupted state of histone H3R2me2a modification, which subsequently influences changes in chromatin accessibility mediated by the Sting protein. Ultimately, this leads to the abnormal activation of Ifitm3 signalling, thereby inducing osteoblast senescence and promoting the development of osteoporosis. This study provides novel theoretical insights and potential intervention targets for understanding the molecular mechanisms underlying osteoblast senescence. We anticipate offering fresh perspectives on the epigenetic mechanisms of bone ageing and identifying novel potential intervention strategies and epigenetic therapeutic targets for delaying or reversing age‐related bone diseases such as osteoporosis.

## 2 Materials and Methods

### 2.1 Osteoblast Culture In Vitro

This study utilized neonatal mouse skulls as the source of bone cells, with experimental samples prepared through the following steps: First, euthanized mice underwent meticulous craniectomy, followed by thorough removal of adherent soft tissue and debris. The skull was then fragmented into small pieces using a grinding disc or surgical knife to facilitate subsequent enzymatic digestion. The skull fragments were placed in a digestion solution containing 0.25% trypsin and 0.05% bile salts, then incubated at 37°C on a rocking incubator for 12–16 h to promote osteocyte release. After digestion, filter the cell suspension through fine mesh or gauze to obtain the pellet. Wash with phosphate‐buffered saline (PBS) and centrifuge to purify the osteoblasts. Finally, place the isolated osteoblasts in suitable culture medium for expansion, laying the foundation for subsequent cellular experiments and research.

### 2.2 Micro‐CT

All experiments involving micro‐CT adhere to the recommended standards of the American Society for Bone and Mineral Research (ASBMR). The brief workflow is as follows: After isolating mouse femurs, they were fixed in 4% paraformaldehyde for 48 h, then stored in 75% ethanol. Images were acquired using the Hiscan XM micro‐CT scanner (manufactured by Suzhou Hiscan Information Technology Co. Ltd.) at a resolution of 25 μm, with X‐ray tube parameters set to 80 kV and 100 μA. Scanning employed 0.5‐degree rotational increments covering a 360‐degree angular range, with a single exposure time of 50 milliseconds. Image reconstruction was performed using Hiscan Reconstruct software (Version 3.0, Suzhou Hiscan Information Technology Co. Ltd.), while evaluation was completed via Hiscan Analyzer software (Version 3.0, Suzhou Hiscan Information Technology Co. Ltd.).

### 2.3 Histology

Femoral bone tissue from different treatment groups was preserved in 4% paraformaldehyde solution for 48 h, followed by decalcification in 10% EDTA solution for three weeks. The treated tissue was dehydrated, embedded in paraffin, and sectioned into 5‐μm‐thick slices. Cellular and tissue morphology was examined in the paraffin sections using haematoxylin and eosin (H&E) staining and immunohistochemical techniques.

### 2.4 Immunohistological Staining

Immunohistochemical Staining Paraffin sections underwent standard dewaxing and rehydration followed by antigen retrieval and blocking with normal serum. Subsequently, sections were incubated with anti‐Prmt6 (15395‐1‐AP, 1:1000, Proteintech, Wuhan, China), anti‐H3Rme2a (2411036, 1:1000, Epigentek, America, NewYork), anti‐Sting (A23923, 1:1000, ABclonal, Wuhan, China), anti‐Ifitm3 (A13070, 1:1000, ABclonal, Wuhan, China) antibodies, and then HRP labelled secondary antibody was incubated at room temperature for 60 min. Colour was developed by incubating with the chromogen 3,3′‐diaminobenzidine (DAB) tetrahydrochloride, followed by counterstaining with haematoxylin. Semiquantitative analysis was performed using ImageJ software.

### 2.5 RNA Extraction and qPCR

RNA was extracted from cells using an RNA extraction kit (R0027, Beyotime, Shanghai, China) and converted into complementary DNA (cDNA) with the Evo M‐MLV reverse transcription kit (R211‐01, Vazyme, Nanjing, China). Hieff qPCR SYBR Green Master Mix (Low Rox Plus) (11202ES03, Yeasen, Shanghai, China) combined with the forward and reverse primers were used for performing RT‐qPCR to measure the mRNA expression of genes. The Gapdh/β‐actin gene was used to normalize other genes, and the values were calculated using the 2^−∆∆CT^ method. Primer sequences are detailed in Table S1.

### 2.6 Western Blot

Extract cellular or tissue proteins using RIPA lysis buffer containing protease inhibitors (P0013, Beyotime, Shanghai, China). Determine protein concentration using the Enhanced BCA Protein Quantification Kit (P0009, Beyotime, Shanghai, China), and separate samples via SDS‐PAGE electrophoresis. After transferring proteins to PVDF membranes (Merck Millipore, USA). Subsequently, incubate overnight at 4°C with primary antibodies, including: anti‐β‐actin (AC026, ABclonal, 1:50,000, Wuhan, China), anti‐Prmt6 (15395‐1‐AP, 1:1000, Proteintech, Wuhan, China), anti‐H3Rme2a (2411036, 1:1000, Epigentek, America, NewYork), anti‐Sting (A23923, 1:1000, ABclonal, Wuhan, China), anti‐Ifitm3 (A13070, 1:1000, ABclonal, Wuhan, China), anti‐P16 (A0262, 1:1000, ABclonal, Wuhan, China), anti‐P21 (A1483, ABclonal, 1:1000, Wuhan, China), anti‐Gpx4 (A11243, 1:1000, ABclonal, Wuhan, China), etc. After washing the membrane three times with phosphate‐buffered saline with Tween 20 (PBST), we used HRP‐conjugated goat anti‐rabbit antibodies (1:10,000) to bind to the primary antibody on the membranes. After washing the membranes three more times, we developed them using an ECL Super Kit (RM02867, ABclonal, Wuhan, China) under a Chemiluminescence Imager.

### 2.7 Cell Senescence Induction and SA‐β‐Gal Staining

Cell Senescence Induction and SA‐β‐Gal Staining: For hydrogen peroxide treatment, add 200 μM hydrogen peroxide to osteoblasts and replace the medium after 2 h. Subsequently, perform SA‐β‐galactosidase staining using the Senescence‐Associated β‐Galactosidase Detection Kit (C0602, Beyotime, Shanghai, China) according to the manufacturer's instructions. After seeding osteoblasts onto culture plates, remove the medium and fix cells at room temperature for 15 min using the provided fixative solution in each well. Subsequently, wash cells twice with PBS and incubate with freshly prepared SA‐β‐Gal staining solution for 16–18 h at 37°C under low CO_2_ and in the dark.

### 2.8 Immunofluorescence

Cells were fixed with 4% paraformaldehyde at 4°C for 15 min, followed by incubation in Triton X‐100 and 5% goat serum. Triton X‐100 and 5% goat serum (P0096, Beyotime, Shanghai, China), followed by overnight incubation at 4°C with the following primary antibodies: anti‐Prmt6 (15395‐1‐AP, 1:1000, Proteintech, Wuhan, China), anti‐H3Rme2a (2411036, 1:1000, Epigentek, America, NewYork), anti‐Sting (A23923, 1:1000, ABclonal, Wuhan, China), anti‐Ifitm3 (A13070, 1:1000, ABclonal, Wuhan, China), anti‐P16 (A0262, 1:1000, ABclonal, Wuhan, China), anti‐P21 (A1483, ABclonal, 1:1000, Wuhan, China), anti‐Alp (68719‐1‐AP, 1:1000, Proteintech, Wuhan, China), anti‐Ocn (23418‐1‐AP, 1:500, Proteintech, Wuhan, China), anti‐Runx2 (20700‐1‐AP, 1:1000, Proteintech, Wuhan, China), followed by fluorescently labelled secondary antibodies. Nuclear counterstaining was performed using DAPI (4′,6‐diaminotoluidine‐2‐phenylindole). Semi‐quantitative evaluation of staining was conducted using ImageJ software version 1.8.0. Integrated optical densities were normalized to regions of interest for calculating average fluorescence intensity.

### 2.9 Animal Husbandry and Material Collection

The animal experiment protocol was approved by the Tongji University Committee for the Protection and Use of Laboratory Animals. Ethics Review Number (Nọ): TJBB04124401. This study utilized female C57BL/6 mice sourced from Shanghai SLAC Laboratory Animal Co Ltd. All animals were housed under standard specific pathogen‐free (SPF) conditions at 22°C ± 2°C, 50% ± 10% relative humidity, with a 12‐h light/dark cycle. They had free access to standard rodent chow and sterile drinking water, and the animal population was maintained through natural mating and breeding. To simulate the natural ageing process and obtain samples from different life stages, three key observation time points were established. Femoral bones were harvested from offspring mice at 2, 12, and 24 months post‐birth. Mice were euthanized using an overdose of anaesthetic (e.g., sodium pentobarbital). After confirming cessation of respiration and heartbeat, the abdomen and thigh muscles were exposed. The hip and knee joints were isolated, and soft tissues were gently removed after complete dissection to preserve the periosteum and both ends of the bone structure. Specimens were fixed in 4% paraformaldehyde.

### 2.10 RNA‐Seq

After raw sequencing data were acquired and the reference genome was prepared, RNA‐seq analysis was performed following a standard pipeline as described below. Briefly, raw reads were subjected to quality control and preprocessing with fastq software to eliminate sequencing adapters and low‐quality reads, yielding clean reads for downstream analyses. Clean reads were mapped to the reference genome using HISAT2, and Stringtie was applied to quantify gene/transcript expression to construct the expression matrix. Differential expression analysis was carried out via DESeq2 based on available biological replicates. Genes with ∣log_2_FC∣ ≥ 1 and *p*_adj_ < 0.05 were defined as significantly differentially expressed genes (DEGs). Subsequently, GO functional enrichment and KEGG pathway enrichment analyses were implemented on DEGs to annotate their biological functions, cellular locations and involved signalling pathways, laying a biological foundation for further mechanistic exploration.

### 2.11 Statistical Analysis

In this study, all data analysis was performed using GraphPad Prism version 10.4.2 to ensure accuracy and consistency in data processing and statistical analysis. Each independent experiment was repeated at least three times to guarantee the reliability and reproducibility of the results. For statistical comparisons, we employed unpaired *t*‐tests and one‐way analysis of variance (ANOVA) to assess the significance of differences between experimental groups. All quantitative data are presented as mean ± standard deviation (SD). A *p*‐value < 0.05 was set as the criterion for statistical significance.

## 3 Results

### 3.1 Establishing Osteoblast Oxidative Stress‐Induced Senescence Models and Replicative Senescence Models

**FIGURE 1:**
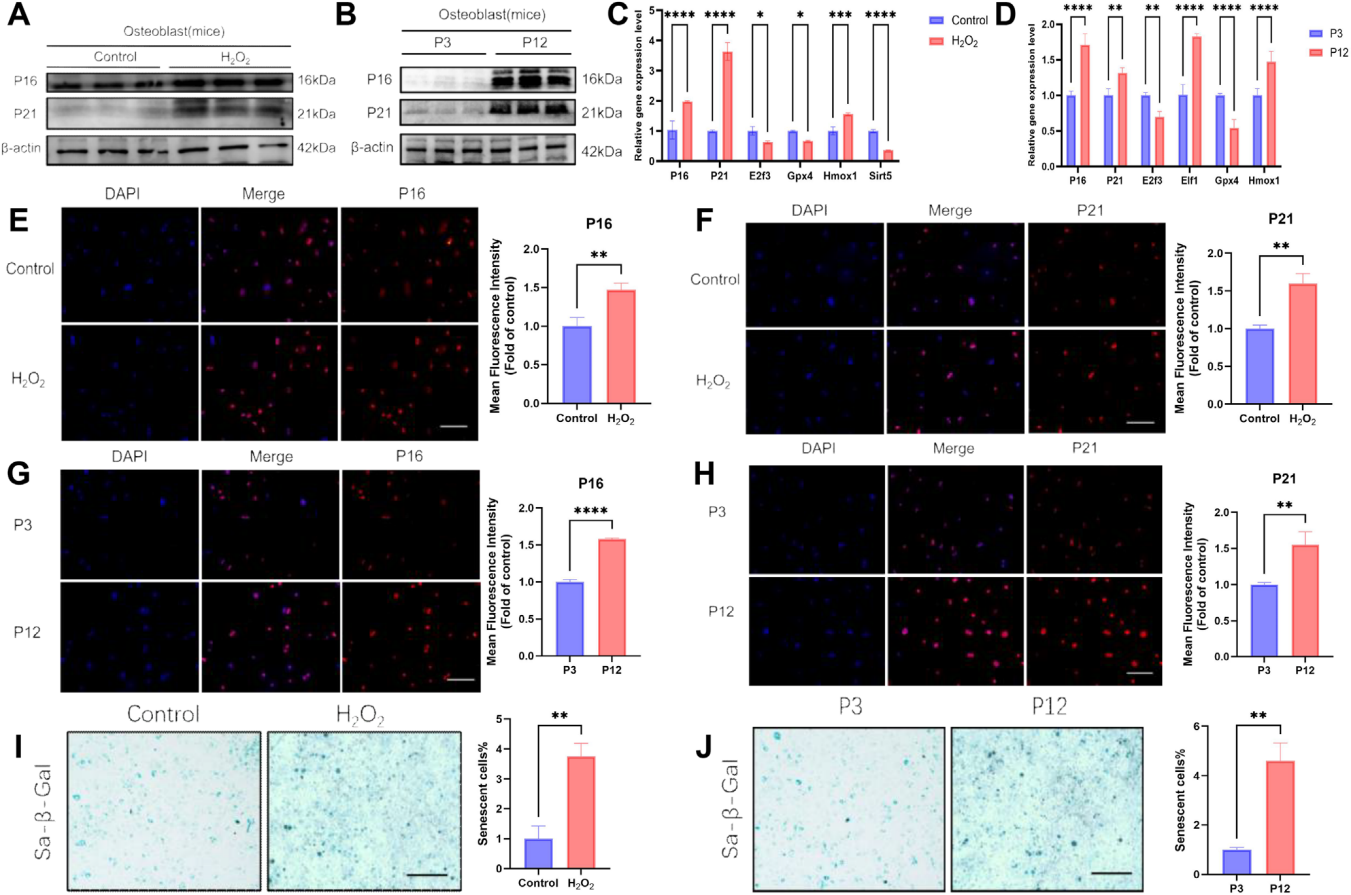
Establishing osteoblast oxidative stress‐induced senescence models and replicative senescence models. (A, B) Western blot analysis of P16 and P21 protein levels in H₂O₂‐induced osteoblast oxidative stress ageing model and replicative ageing model. (C) qPCR analysis of mRNA levels of P16, P21, Homx1, E2f3, Gpx4, and Sirt5 in H₂O₂‐induced osteoblast oxidative stress ageing model. (D) qPCR detection of mRNA levels for P16, P21, Elf1, Homx1, E2f3, and Gpx4 in the replicative senescence model. (E, F) Immunofluorescence detection of P16 and P21 expression levels in the H₂O₂‐induced osteoblast oxidative stress senescence model. Scale bar = 50 μm. (G, H) Immunofluorescence detection of P16 and P21 expression levels in the replicative senescence model. Scale bar = 50 μm. (I, J) Galactosidase staining shows the percentage of SA‐β‐gal positive cells increases with cellular ageing. Scale bar = 100 μm. Statistical analysis was performed using one‐way ANOVA followed by Tukey's multiple comparisons test. *p < 0.05, **p < 0.01, ***p < 0.001, ****p < 0.0001; ns, not significant.

We induced osteoblast senescence through two classical approaches: natural passage and H_2_O_2_ (200 μM) stimulation, to investigate the involvement of relevant molecules in osteoblast senescence. First, an oxidative stress‐induced senescence model was established in osteoblasts using H_2_O_2_. Western blot results showed that compared with the control group, the H_2_O_2_‐treated group exhibited increased expression of P16 and P21 proteins (Figure 1A). qPCR results showed that compared with the control group, the expression levels of P16, P21, and Homx1 were elevated in the H_2_O_2_ treatment group, while the expression levels of E2f3, Gpx4, and Sirt5 were decreased (Figure 1C). Subsequently, immunofluorescence experiments further validated these findings, revealing a significant increase in P16 and P21 expression in osteoblasts following H_2_O_2_ addition (Figure 1E,F). Concurrently, galactosidase staining indicated an elevated proportion of SA‐β‐gal positive cells (Figure 1I). Collectively, these results confirm the successful establishment of an oxidative stress‐induced senescence model in osteoblasts. A replicative senescence model of osteoblasts was established through natural cell passaging. Western blot results showed that P16 and P21 protein expression levels increased with the number of osteoblast passages (Figure 1B). qPCR results indicated that expression levels of P16, P21, Elf1, and Homx1 increased, while those of E2f3 and Gpx4 decreased with increasing osteoblast passage numbers (Figure 1D). Subsequently, immunofluorescence experiments further validated these findings, demonstrating a significant increase in P16 and P21 expression with passage number (Figure 1G,H). Concurrently, galactosidase staining indicated an increased proportion of SA‐β‐gal positive cells (Figure 1J). Collectively, these results confirm the successful establishment of a replicative senescence model in osteoblasts.

### 3.2 Prmt6 Expression Is Downregulated in Senescent Osteoblasts and Prmt6 Knockdown Promotes Osteoblast Senescence

**FIGURE 2:**
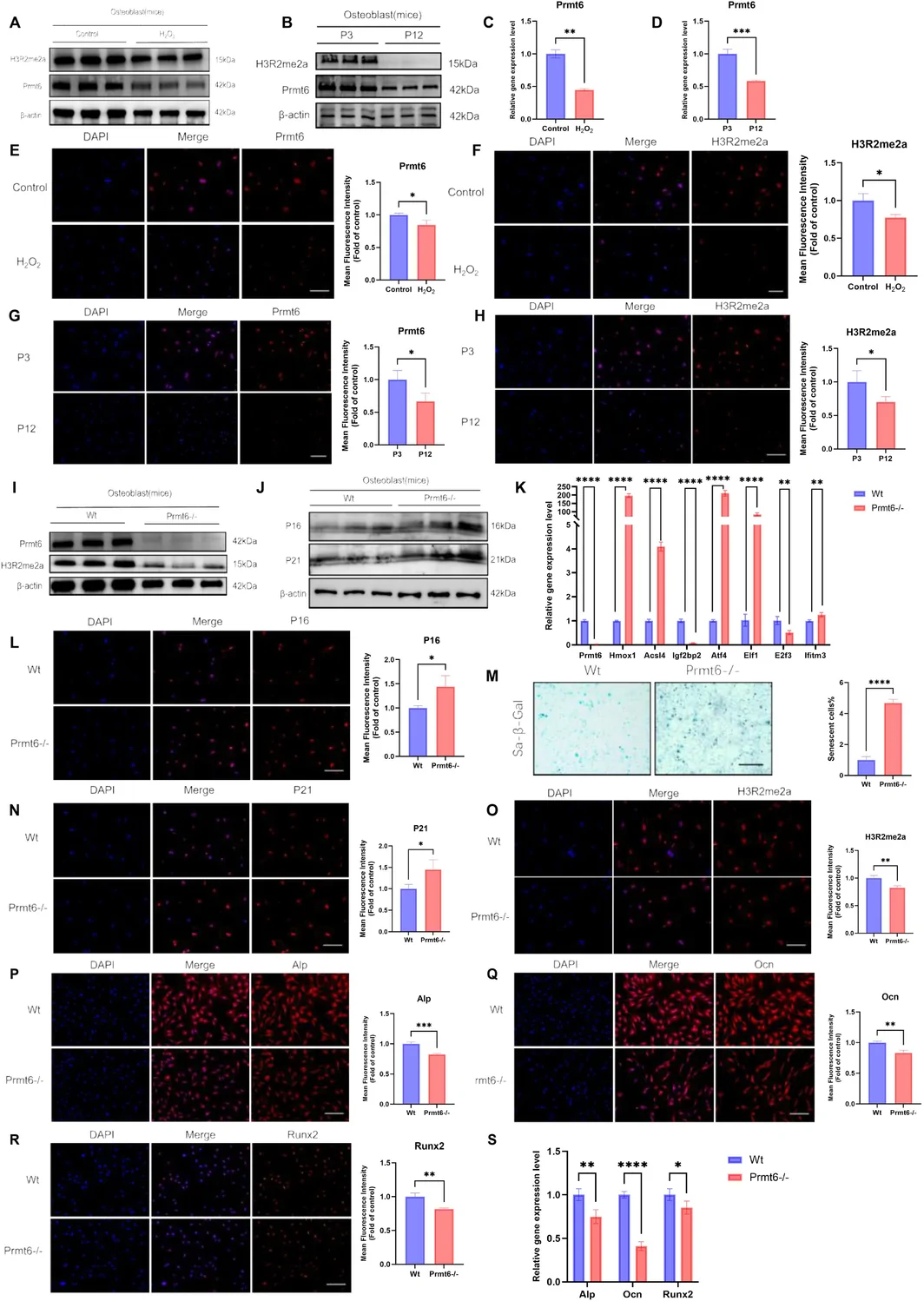
Prmt6 expression is downregulated in senescent osteoblasts and Prmt6 knockdown promotes osteoblast senescence. (A, B) Western Blot analysis of Prmt6 and H3R2me2a protein levels in H₂O₂‐induced osteoblast oxidative stress ageing model and replicative ageing model. (C, D) qPCR analysis of Prmt6 mRNA levels in H₂O₂‐induced osteoblast oxidative stress ageing model and replicative ageing model. (E–H) Immunofluorescence analysis of Prmt6 and H3R2me2a protein levels in both ageing models. Scale bar = 50 μm. (I and J) Western Blot analysis of Prmt6, H3R2me2a, P16, and P21 protein levels in Prmt6 ko osteoblasts. (K) qPCR detection of mRNA levels of Prmt6, Homx1, Acsl4, Igf2bp2, Atf4, Elf1, E2f3, and Ifitm3 in Prmt6 ko osteoblasts. (L, N, O) Immunofluorescence analysis of P16, P21, and H3R2me2a protein levels following Prmt6 knockdown. Scale bar = 50 μm. (M) Percentage of SA‐β‐gal‐positive cells increased with Prmt6 knockdown. Scale bar = 100 μm. (P–R) Immunofluorescence assay to detect the protein levels of Alp, Ocn, and Runx2 in Prmt6‐knockout osteoblasts. (S) qPCR assay to detect the mRNA levels of Alp, Ocn, and Runx2 in Prmt6‐knockout osteoblasts. Statistical analysis was performed using one‐way ANOVA followed by Tukey's multiple comparisons test. *p < 0.05, **p < 0.01, ***p < 0.001, ****p < 0.0001; ns, not significant.

To investigate the expression of Prmt6 in senescent osteoblasts, we employed an H_2_O_2_‐induced oxidative stress model and a replicative senescence model. Western blot analysis revealed that compared to the control group, both Prmt6 expression levels and H3R2me2a protein expression levels were reduced (Figure 2A,B). qPCR results showed that Prmt6 mRNA levels were reduced in H_2_O_2_ treatment group cells compared to the control group (Figure 2C). Additionally, in the replicative senescence model, Prmt6 mRNA levels decreased with increasing passage number of osteoblasts (Figure 2D). Immunofluorescence analysis further confirmed elevated protein expression levels of Prmt6 and H3R2me2a in both senescence models (Figure 2E–H). These results indicate reduced Prmt6 expression in aged osteoblasts. To further validate the biological function of Prmt6 in osteoblasts, we isolated and obtained Prmt6 knockout (Prmt6 ko) osteoblasts from Prmt6 knockout (Prmt6−/−) mice. Western blot results showed significantly reduced levels of Prmt6 and H3R2me2a proteins in osteoblasts compared to the control group (Figure 2I), confirming successful Prmt6 gene knockout in mouse osteoblasts. Compared to the control group, protein expression of P16 and P21 increased in knockout osteoblasts, indicating that Prmt6 knockout promotes osteoblast senescence (Figure 2J). qPCR experiments revealed significantly elevated mRNA levels of Prmt6, Hmox1, Acsl4, Atf4, Elf1, and Ifitm3 in Prmt6 ko osteoblasts, alongside markedly reduced levels of Igf2bp2 and E2f3, indicating that Prmt6 knockdown promotes osteoblast senescence (Figure 2K). Immunofluorescence experiments further confirmed that the fluorescence intensities of P16 and P21 were significantly higher in the knockdown group than in the control group, while H3R2me2a fluorescence intensity was lower than in the control group (Figure 2L,N,O). Additionally, galactosidase staining indicated an increased proportion of SA‐β‐gal positive cells in the knockdown group (Figure 2M). Furthermore, immunofluorescence staining results showed that the fluorescence intensity of Alp, Ocn, and Runx2 in cells from the Prmt6 knockout (Prmt6 KO) group was significantly weaker than that in the control group, suggesting decreased expression levels of these proteins (Figure 2P–R). qPCR analysis showed that, compared with the control group, the mRNA levels of the osteogenic differentiation marker genes Alp, Ocn, and Runx2 were all significantly reduced in Prmt6‐knockout osteoblasts (Figure 2S). These results indicate that Prmt6 knockdown promotes osteoblast senescence.

### 3.3 Comprehensive Analysis of Gene Expression Differences Between Wild‐Type and Prmt6 Knockout Models

**FIGURE 3:**
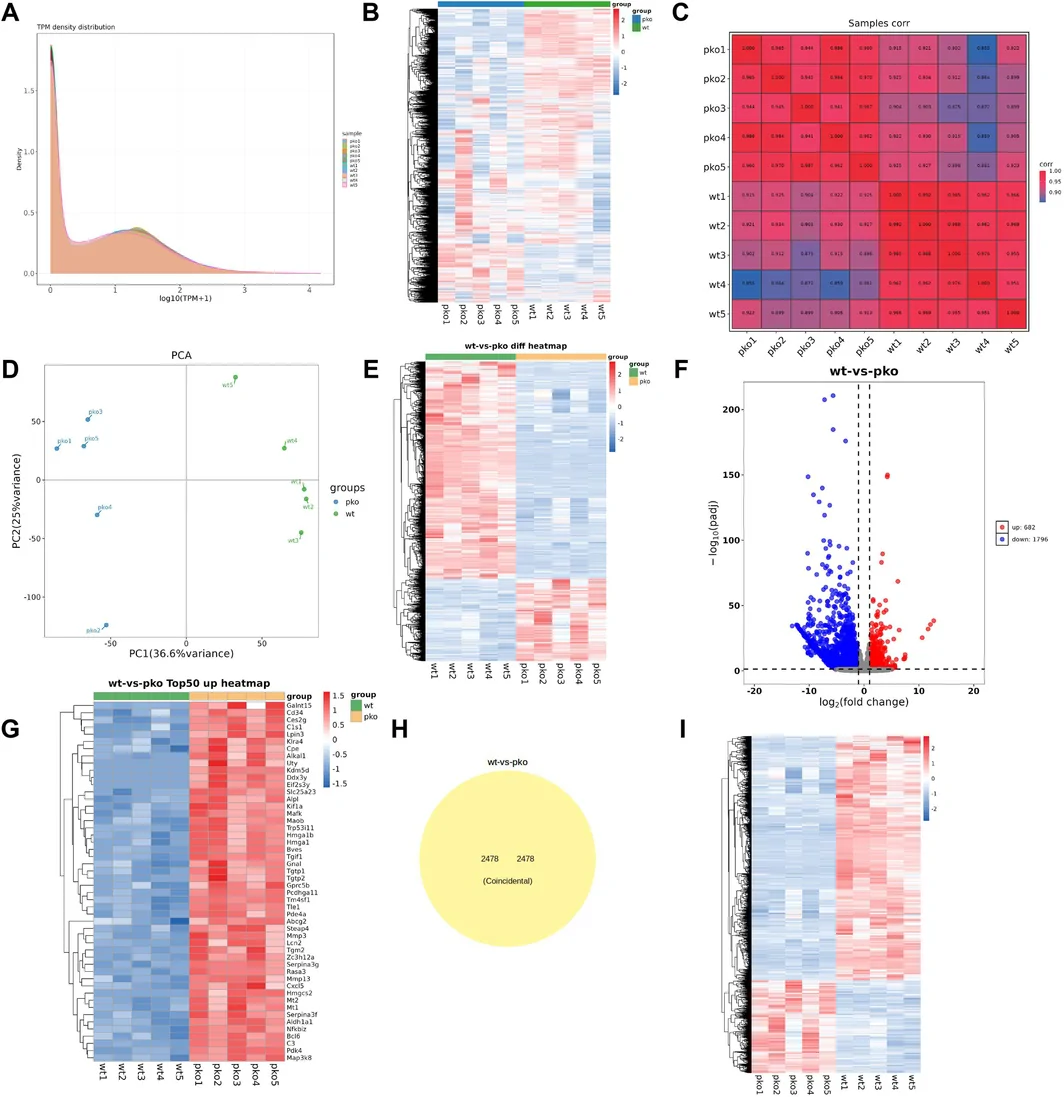
Comprehensive analysis of gene expression differences between wild‐type and Prmt6 knockout models. (A) Gene expression level density plot. (B) Gene expression level heatmap. (C) Sample correlation heatmap. (D) Principal component analysis (PCA) results plot. (E) Expression heatmap of all differentially expressed genes from DESeq2 or edgeR analysis. (F) Volcano plot showing the distribution of differentially expressed genes for each comparison combination. (G) Expression heatmap of all differentially expressed genes from DESeq2 or edgeR analysis, hierarchically clustered based on expression patterns of the 50 most significant differentially expressed genes (based on corrected *p*‐value or *p*‐value). (H) Co‐expression gene network diagram comparing the experimental group and treatment group with the control group. (I) Clustering heatmap of the union of differentially expressed genes across different groupings. Density distribution plot, sample correlation heatmap and PCA analysis of RNA‐seq data. Each experimental group is clearly distinguished by different colours; *n* = 5 biological replicates per group.

To further investigate the biological function of Prmt6, we performed RNA sequencing. First, the raw data underwent rigorous screening to remove outliers and anomalies. Corrections were then applied for sequencing depth and gene length. After calculating the expression values (TPM) for all genes across each sample, density distribution plots were generated to visualize the distribution of gene expression levels across different samples, as shown below. The horizontal axis represents log10 (TPM + 1), while the vertical axis denotes density. Although slight variations exist between the distribution curves of different samples, the overall trends are similar, indicating that the distribution of gene expression levels across samples exhibits a degree of similarity (Figure 3A). Hierarchical clustering was performed on the expression patterns of all genes, and the clustering results were visualized using a heatmap (Figure 3B). Simultaneously, sample correlations were investigated by calculating intra‐group and inter‐group correlation coefficients based on the TPM values of all genes across samples. A correlation coefficient closer to 1 indicates higher similarity in expression patterns between samples (Figure 3C). Principal component analysis (PCA) was performed on the samples to visualize overall transcriptomic differences between them. In the PCA plot, samples should be dispersed between groups and clustered within groups (Figure 3D). The PCA results showed that control and Prmt6 knockdown group samples were clearly separated along the first principal component axis, indicating that Prmt6 deficiency significantly altered the transcriptional profile of osteoblasts. Intra‐group biological replicates showed high clustering, indicating good reproducibility of the experiment. Further differential analysis was performed by hierarchical clustering of expression patterns for all differentially expressed genes (DESeq2 or edgeR), with heatmaps presented to visualize clustering results (Figure 3E). The volcano plot revealed 2478 differentially expressed genes, including 682 upregulated and 1796 downregulated genes (|log2 FC| > 2, *p*_adj_ < 0.05) (Figure 3F). Hierarchical clustering was performed on the expression patterns of the 50 most significant differentially expressed genes based on corrected *p*‐values or *p*‐values, with the clustering results visualized using a heatmap (Figure 3G). These genes exhibited significant expression differences between the two groups. To further validate the functional enrichment characteristics of differentially expressed genes and to demonstrate the overlap of differentially expressed genes across different comparison sets, a Venn diagram was used to identify 2478 differentially expressed genes that were either common to or unique to specific comparison sets (Figure 3H). All differentially expressed genes from all comparison sets were combined into a union, and the union genes underwent cluster analysis (Figure 3I). In summary, Prmt6 knockout induced significant changes in gene expression that were consistent across different samples. These findings provide important clues for understanding the function and impact of the knocked‐out gene and may guide subsequent biological research and applications.

### 3.4 Three Enrichment Analyses Reveal Functional Characteristics of Multiple Cellular Pathways and Differentially Expressed Genes Following Prmt6 Knockout

**FIGURE 4:**
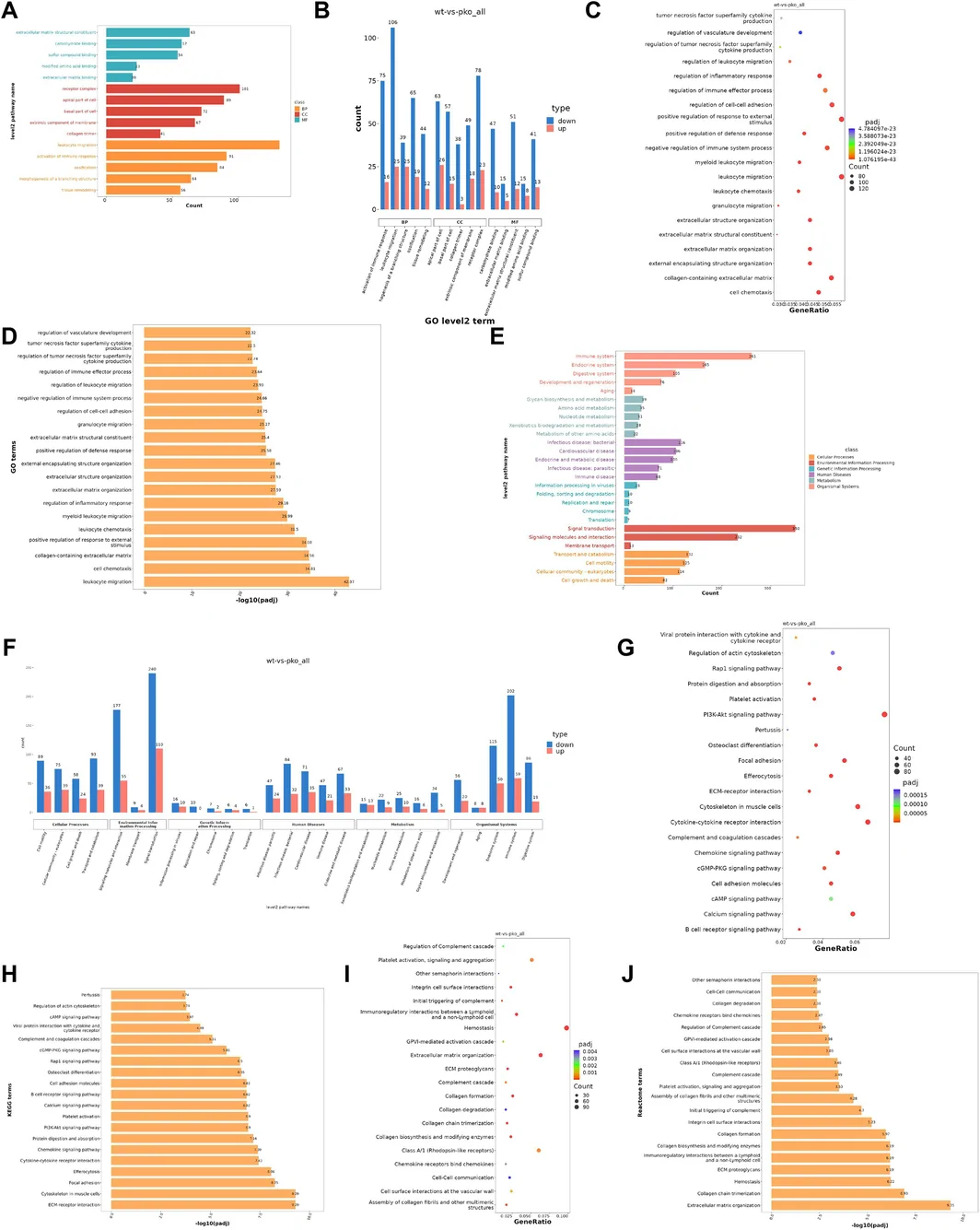
Three enrichment analyses reveal functional characteristics of multiple cellular pathways and differentially expressed genes following Prmt6 knockout. (A) From the GO enrichment analysis results, select the top five most significant terms at level 2 across different classifications (BP, CC, MF) and display them in a bar chart. (B) Based on the GO enrichment analysis results, select the top five most significant level 2 terms under each category (BP, CC, MF) and generate bar charts for presentation, grouped by upregulated and downregulated differentially expressed genes. (C) From the GO enrichment analysis results, select the top 20 most significant terms and plot them as a scatter plot for visualization. (D) From the GO enrichment analysis results, select the top 20 most significant terms (with the smallest *p*_adj_ values) and plot them as a bar chart for visualization. (E) From the KEGG enrichment analysis results, select the top five most significant pathways at level 2 under different classifications and display them as a bar chart. (F) From the KEGG enrichment analysis results, select the top five most significant pathways at level 2 under different classifications and display them as a bar chart. (G) From the KEGG enrichment analysis results, select the top 20 most significant terms and display them using a scatter plot. (H) From the KEGG enrichment analysis results, select the top 20 most significant pathways (with the smallest *p*_adj_) and display them using a bar chart. (I) From the Reactome enrichment analysis results, select the top 20 most significant (smallest *p*_adj_) terms and display them in a scatter plot. (J) From the Reactome enrichment analysis results, select the top 20 most significant (smallest *p*_adj_) pathways and display them in a bar chart.

After identifying differentially expressed genes through gene expression analysis, it is essential to further elucidate their biological functions. This study employed the clusterProfiler software to perform GO functional enrichment analysis and KEGG pathway enrichment analysis on the differentially expressed gene sets. The enrichment analysis results were conducted for all differentially expressed gene sets, up‐regulated gene sets, and down‐regulated gene sets within each differential comparison combination. GO enrichment analysis revealed that differentially expressed genes were concentrated in three major modules: ‘Collagen trimers/matrix structural components’, ‘Immune cell migration’, and ‘Ossification and branching morphogenesis’. This suggests that osteoporosis arises from a synergistic imbalance involving suppressed osteoblast differentiation, enhanced osteoclast recruitment, and disrupted extracellular matrix remodelling (Figure 4A). The GO functional annotation diagram reveals that osteogenesis‐related processes (bone development, extracellular matrix formation, cell adhesion, etc.) are significantly downregulated in the osteoporosis model, while inflammatory responses and cytokine activity are upregulated. This suggests that suppressed bone formation, structural bone destruction, and chronic low‐grade inflammation jointly drive osteoporosis development (Figure 4B). The GO enrichment analysis scatter plot reveals that osteoporosis‐associated genes cluster along the ‘TNF‐α production‐leukocyte migration‐vascular development‐collagen matrix’ axis (*p*_adj_ < 1 × 10^−23^), suggesting that hyperactive osteoclastic inflammation, insufficient osteoblastic blood supply, and impaired matrix assembly collectively drive bone loss (Figure 4C). Higher –log_10_(*p*_adj_) values indicate more significant enrichment. The top four enriched pathways—TNF‐α production, vascular regulation, myeloid cell migration, and collagen matrix—suggest osteoporosis is driven by simultaneous dysregulation across the ‘inflammation‐vascular‐matrix’ triad (Figure 4D). KEGG pathway enrichment analysis further reveals that the molecular basis of osteoporosis involves a triple network imbalance encompassing immune, endocrine, and cellular fate regulation: excessive activation of immune signalling, disrupted regulation of osteoblast–osteoclast fate, and superimposed endocrine‐metabolic abnormalities collectively drive bone mass loss and vascular‐bone microstructural disruption (Figure 4E). KEGG upregulation/downregulation classification revealed that osteoporosis‐associated differentially expressed genes were predominantly downregulated. Downregulated entries significantly outnumbered upregulated ones in immune, cell movement, and endocrine metabolism pathways, suggesting suppressed osteoblastic vascular homeostasis signalling coupled with enhanced osteoclastic inflammatory signalling, leading to bone mass loss (Figure 4F). KEGG enrichment analysis revealed that osteoporosis‐associated differentially expressed genes significantly clustered in pathways including osteoclast differentiation, cytokine‐receptor interactions, PI3K‐Akt signalling, and Rap1 signalling. These genes were co‐downregulated with actin cytoskeleton remodelling and ECM‐receptor interactions, suggesting suppressed osteoblast cytoskeleton‐adhesion‐survival signalling, enhanced osteoclast differentiation drive, and subsequent bone mass loss (Figure 4G). The KEGG enrichment bar chart points from high to low toward the cascade ‘osteoclast differentiation‐cytokine interactions‐PI3K‐Akt‐actin cytoskeleton‐ECM receptors’, revealing identical results (Figure 4H). Reactome enrichment analysis revealed that differentially expressed genes were significantly enriched in classical pathways such as extracellular matrix organization, collagen formation, and skeletal system development, while also involving negative regulation of inflammatory responses and immune response balance (Figure 4I,J). The above analyses systematically elucidated the functional characteristics and potential biological significance of multiple cellular pathways and differentially expressed genes following Prmt6 knockout, providing a theoretical basis for subsequent functional validation.

### 3.5 Sting Expression Is Elevated in Senescent Osteoblasts and Sting Knockdown Inhibits Osteoblast Senescence

**FIGURE 5:**
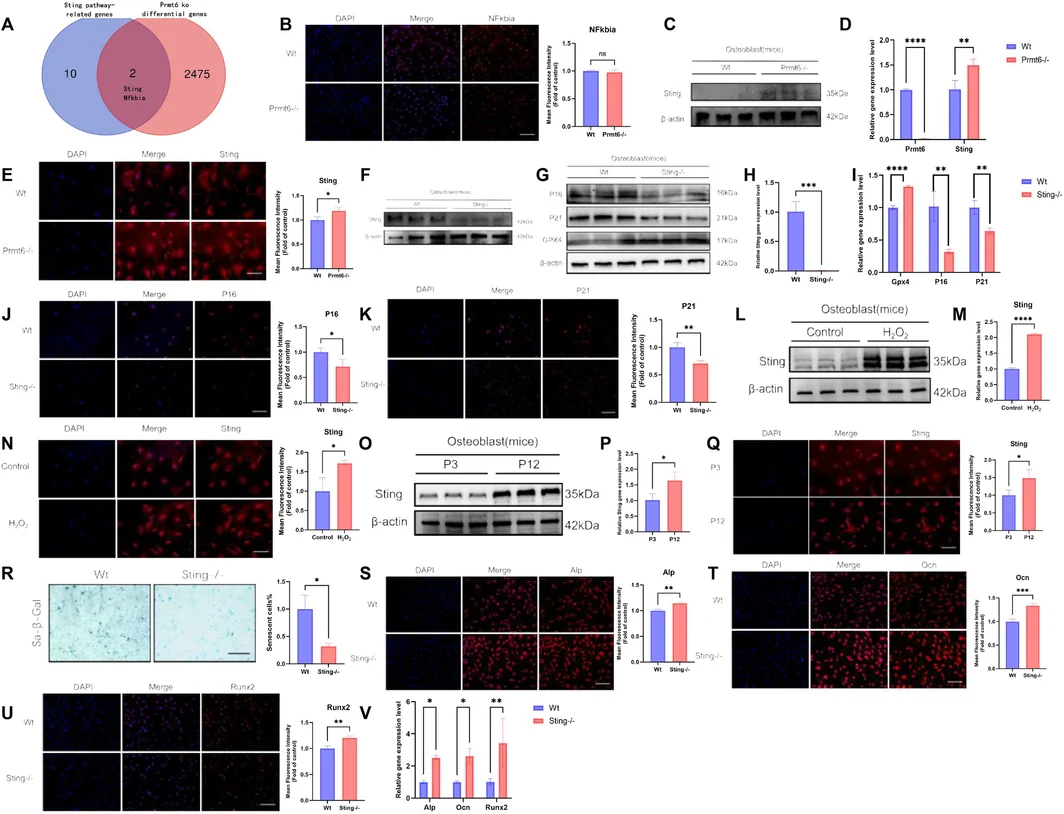
Sting expression is elevated in senescent osteoblasts, and Sting knockdown inhibits osteoblast senescence. (A) Venn diagram showing the overlap between differentially expressed genes and Sting‐related genes. (B) Immunofluorescence assay to detect NFkbia protein levels in Prmt6‐ko osteoblasts. (C–E) Western blot, qPCR, and immunofluorescence assays detecting Sting expression levels in Prmt6 ko osteoblasts. Scale bar = 50 μm. (F and G) Western blot detecting protein levels of Sting, P16, P21, and Gpx4 after Sting gene knockdown. (H and I) qPCR analysis of Sting knockdown efficiency and mRNA levels of P16, P21, and Gpx4. (J and K) Immunofluorescence experiments showing changes in P16 and P21 fluorescence intensity in osteoblasts after Sting knockdown. Scale bar = 50 μm. (L–N) Western Blot, qPCR, and immunofluorescence assays detecting Sting protein and mRNA levels in osteoblast oxidative stress models. Scale bar = 50 μm. (O–Q) Western Blot, qPCR, and immunofluorescence assays detecting Sting protein and mRNA levels in osteoblasts from replicative ageing models. Scale bar = 50 μm. (R) The percentage of SA‐β‐gal‐positive cells decreased with Sting knockdown. Scale bar = 100 μm. (S–U) Immunofluorescence assay to detect the protein levels of Alp, Ocn, and Runx2 in Sting‐knockout osteoblasts. (V) qPCR assay to detect the mRNA levels of Alp, Ocn, and Runx2 in Sting‐knockout osteoblasts. Statistical analysis was performed using one‐way ANOVA followed by Tukey's multiple comparisons test. *p < 0.05, **p < 0.01, ***p < 0.001, ****p < 0.0001; ns, not significant.

The intersection of differentially expressed genes identified by RNA‐seq and genes associated with the Sting pathway yielded two differentially expressed genes: Sting and NFkbia (Figure 5A). Through a Venn diagram analysis, both Sting and Nfkbia were identified as candidate target genes. Following Prmt6 knockout, there were no significant changes in Nfkbia mRNA expression (Figure 5B). Based on these results, this study excluded Nfkbia and prioritized the Sting gene—which is stably regulated by Prmt6—for further mechanistic investigation. Research indicates that the cGAS‐STING signalling pathway mediating DNA immune recognition is a key driver of chronic inflammation and age‐related functional decline [38]. The release of mitochondrial DNA promotes cGAS‐STING activation and accelerates senescence in post‐mitotic muscle cells [39], suggesting that Sting plays a significant role in osteoblast senescence. Western blot, qPCR, and immunofluorescence analysis revealed that Prmt6 knockdown led to increased levels of both Sting protein and mRNA, indicating that Prmt6 deficiency significantly upregulates Sting expression (Figure 5C–E). To further validate the biological function of Sting in osteoblasts, we isolated and obtained Sting knockout (Sting ko) osteoblasts from Sting knockout (Sting−/−) mice (Figure 5F). Western blot analysis revealed reduced protein levels of the senescence markers P16 and P21, along with increased Gpx4 expression, in osteoblasts with Sting knockdown (Figure 5G). qPCR experiments similarly revealed the same mRNA level trends (Figure 5H,I). In immunofluorescence assays, both P16 and P21 protein levels were lower in Sting ko osteoblasts compared to the control group (Figure 5J,K), indicating that osteoblasts are more prone to senescence following Sting gene knockdown. In the H_2_O_2_‐induced oxidative stress‐induced senescence model of osteoblasts, Sting protein levels, mRNA levels, and immunofluorescence intensity were elevated compared to the control group (Figure 5L–N). Additionally, in the replicative senescence model, both Sting protein levels and mRNA levels increased in the 12th‐pass osteoblasts compared to the 3rd‐pass osteoblasts as the passage number increased (Figure 5O–Q). Furthermore, galactosidase staining indicated a reduced proportion of SA‐β‐gal positive cells in the Sting knockdown group (Figure 5R). Furthermore, immunofluorescence staining results showed that the fluorescence intensity of Alp, Ocn, and Runx2 in cells from the Sting knockout (Sting KO) group was significantly stronger than that in the control group, suggesting elevated expression levels of these proteins (Figure 5S–U). qPCR analysis showed that, compared with the control group, the mRNA levels of the osteogenic differentiation marker genes Alp, Ocn, and Runx2 were all significantly elevated in Sting‐knockout osteoblasts (Figure 5V). In summary, Sting is highly expressed in senescent osteoblasts, and inhibiting Sting expression effectively suppresses osteoblast senescence, providing a novel potential target for preventing and treating osteoblast senescence‐related diseases.

### 3.6 Ifitm3 Is Highly Expressed in Senescent Osteoblasts and Ifitm3 Knockdown Inhibits Osteoblast Senescence

**FIGURE 6:**
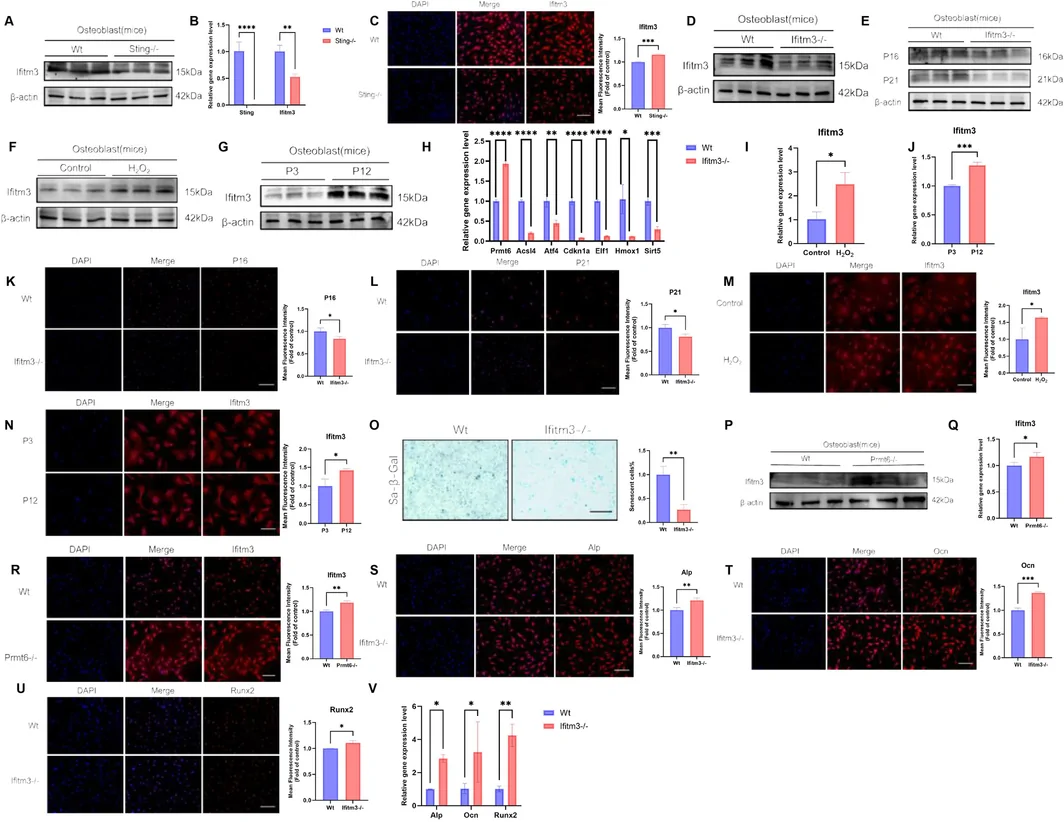
Ifitm3 is highly expressed in senescent osteoblasts and Ifitm3 ko inhibits osteoblast senescence. (A–C) Western Blot, qPCR, and immunofluorescence assays detected Ifitm3 expression levels in Prmt6ko osteoblasts. (D and E) Western Blot detected protein levels of Ifitm3, P16, and P21 after Sting knockdown. (F) Western Blot detected Ifitm3 protein levels in an osteoblast oxidative stress model. (G) Western Blot detection of Ifitm3 protein expression levels in replicative senescence models. (H) Detection of mRNA levels for Prmt6, Acsl4, Atf4, Cdkn1a, Elf1, Hmox1, and Sirt5 in Ifitm3 ko osteoblasts. (I and J) qPCR detection of Ifitm3 mRNA expression levels in two senescence models. (K and L) Immunofluorescence analysis of P16 and P21 protein levels in Ifitm3 ko osteoblasts. Scale bar = 50 μm. (M and N) Immunofluorescence analysis of Ifitm3 protein levels in two ageing models. Scale bar = 50 μm. (O) Percentage of SA‐β‐gal positive cells decreased with Ifitm3 knockdown. Scale bar = 100 μm. (P–R) Western Blot, qPCR, and immunofluorescence assays detected Ifitm3 protein and mRNA levels in Prmt6 ko osteoblasts. Scale bar = 50 μm. (S–U) Immunofluorescence assays were performed to detect the protein levels of Alp, Ocn, and Runx2 in Ifitm3‐knockout osteoblasts. (V) qPCR assays were performed to detect the mRNA levels of Alp, Ocn, and Runx2 in Ifitm3‐knockout osteoblasts. Statistical analysis was performed using one‐way ANOVA followed by Tukey's multiple comparisons test. *p < 0.05, **p < 0.01, ***p < 0.001, ****p < 0.0001; ns, not significant.

To investigate the effect of Sting on Ifitm3 and the relationship between Ifitm3 and osteoblast senescence, we conducted the following experiments. Western blot analysis revealed a significant reduction in Ifitm3 expression following Sting knockdown, suggesting Sting may positively regulate Ifitm3 expression (Figure 6A). qPCR further validated this finding, demonstrating a marked decrease in Ifitm3 mRNA levels after Sting silencing (Figure 6B). Immunofluorescence staining also demonstrated that the fluorescence signal of Ifitm3 in senescent osteoblasts was attenuated following Sting knockdown (Figure 6C). These data collectively indicate that the high expression of Ifitm3 in senescent osteoblasts depends on the activation of the Sting pathway, suggesting a potential functional association between the two in cellular senescence. To further validate the biological function of Ifitm3 in osteoblasts, we isolated and obtained Ifitm3 knockout (Ifitm3 ko) osteoblasts from Ifitm3 knockout (Ifitm3−/−) mice (Figure 6D). Western blot analysis revealed reduced levels of the senescence markers P16 and P21 in Ifitm3 knockout osteoblasts (Figure 6E), indicating that Ifitm3 knockout partially suppresses osteoblast senescence. In an H_2_O_2_‐induced oxidative stress model of osteoblasts, Ifitm3 protein levels were elevated compared to controls (Figure 6F). Furthermore, in a replicative senescence model, Ifitm3 protein levels increased with passage number compared to the control group (Figure 6G). qPCR results showed that Ifitm3 knockdown increased mRNA levels of Prmt6 in osteoblasts, while decreasing mRNA levels of Acsl4, Atf4, Cdkn1a, Elf1, Hmox1, and Sirt5 (Figure 6H). In the treated group of the oxidative stress model, Ifitm3 mRNA levels were elevated (Figure 6I). Concurrently, in the replicative ageing model, Ifitm3 mRNA levels exhibited a progressive upward trend with increasing passage numbers (Figure 6J). Immunofluorescence analysis revealed decreased protein levels of the ageing markers P16 and P21 in Ifitm3 ko osteoblasts (Figure 6K,L). In the oxidative stress model, Ifitm3 protein expression was elevated compared to the control group (Figure 6M), and its mRNA levels showed a progressive increase with passage number (Figure 6N). Galactosidase staining indicated a reduced proportion of SA‐β‐gal positive cells in the knockdown group (Figure 6O). These results indicate high Ifitm3 expression in senescent osteoblasts. To investigate the specific mechanism by which Prmt6 regulates Ifitm3 in osteoblast senescence, we further examined Ifitm3 expression changes in Prmt6 ko osteoblasts. Western blot analysis revealed a significant downregulation of Ifitm3 mRNA levels following Prmt6 knockdown (Figure 6P), while Ifitm3 mRNA levels were also markedly reduced (Figure 6Q), suggesting Prmt6 may influence Ifitm3 expression by regulating its transcription or mRNA stability. Immunofluorescence analysis further revealed that Prmt6 knockdown significantly enhanced Ifitm3 expression activity (Figure 6R). These findings collectively demonstrate increased Ifitm3 expression in Prmt6 ko osteoblasts. Furthermore, immunofluorescence staining results showed that the fluorescence intensity of Alp, Ocn, and Runx2 in cells from the Ifitm3 knockout group was significantly stronger than that in the control group, suggesting elevated expression levels of these proteins (Figure 6S–U). qPCR analysis indicated that, compared with the control group, the mRNA levels of the osteogenic differentiation marker genes Alp, Ocn, and Runx2 were all significantly elevated in Ifitm3‐knockout osteoblasts (Figure 6V). In summary, Ifitm3 overexpression depends on Sting pathway activation. Ifitm3 is highly expressed in senescent osteoblasts, and its knockdown inhibits osteoblast senescence.

### 3.7 Prmt6 and H3R2me2a Expression Decreases in Aged Femoral Tissue and Sting and Ifitm3 Expression Increases in Aged Femoral Tissue

**FIGURE 7:**
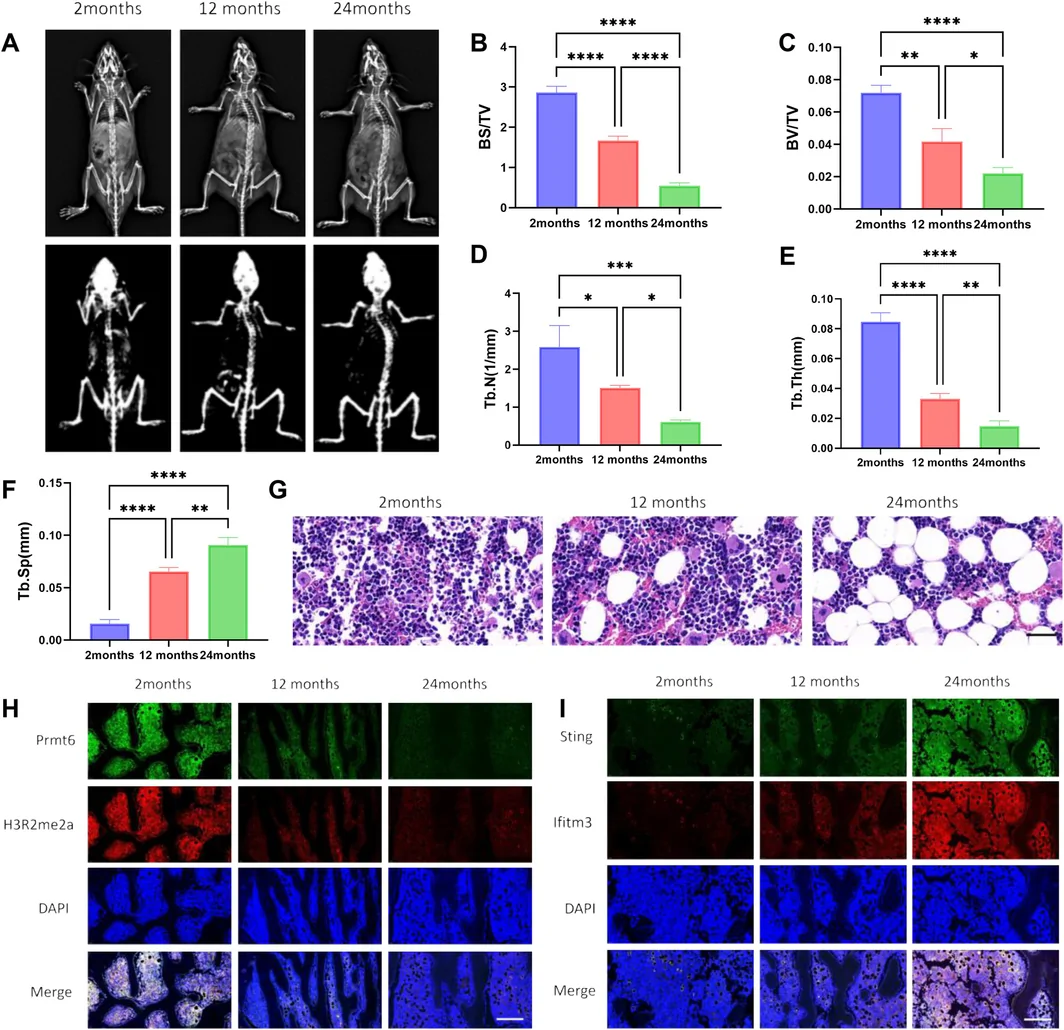
Prmt6 and H3R2me2a expression decreases in aged femoral tissue and Sting and Ifitm3 expression increase in aged femoral tissue. (A) Representative images from micro‐CT analysis of aged mice. (B–F) Micro‐CT analysis of trabecular microarchitecture: BS/TV (B), BV/TV (C), Tb.N (D), Tb.Th (E), and Tb.Sp (F) (*n* = 3). (G) Representative images of HE‐stained tissue sections from aged mice. Scale bar = 20 μm. (H) Representative images of dual immunofluorescence staining for Prmt6 and H3R2me2a in the femurs of aged mice. (I) Representative images of dual immunofluorescence staining for Sting and Ifitm3 in the femurs of aged mice. Scale bar = 50 μm. Statistical analysis was performed using one‐way ANOVA followed by Tukey's multiple comparisons test. *p < 0.05, **p < 0.01, ***p < 0.001, ****p < 0.0001; ns, not significant. BS, bone surface area; BS/TV, bone surface area to tissue volume ratio; BV, volume of bone; BV/TV, bone mass and fraction; Tb.N, tubular bone number; Tb.Sp, tubular bone spacing; Tb.Th, tubular bone thickness; TV, volume of tissue.

To further evaluate the effects of Prmt6, H2R2me2a, Sting, and Ifitm3 on osteoporotic mice, we performed micro‐CT analysis on mice at 2, 12, and 24 months of age, respectively. Micro‐CT analysis revealed that compared to 2‐month‐old mice, both the 12‐month‐old and 24‐month‐old groups exhibited a significant reduction in trabecular number (Figure 7A). In aged mice, BV/TV, BS/TV, Tb.N, and Tb.Th were significantly decreased (Figure 7B–E), while Tb.Sp was significantly increased (Figure 7F). Haematoxylin and eosin staining revealed that in the aged group, the edges of trabeculae in the femoral tissue appeared coarse, with enlarged trabecular spaces and displaced trabeculae indicating fractures. Additionally, there was an increase in vacuoles within the medullary cavity (Figure 7G). These findings confirm that osteoporosis is indeed present in the aged mice used in this study, and that its severity progressively increases with advancing age. At the animal level, we also found that total Prmt6 and H3R2me2a levels in the femurs of aged mice were significantly lower than those in young mice and decreased with increasing age (Figure 7H). Concurrently, Sting expression and Ifitm3 levels were elevated in femoral tissue (Figure 7I). These findings align with trends observed in cellular experiments, further substantiating the association between osteoporosis‐related Prmt6, H3R2me2a, Sting, and Ifitm3 expression and age‐related osteoporosis.

## 4 Discussion

Osteoporosis (OP), a metabolic bone disease prevalent among the elderly, is characterized by reduced bone mineral density and abnormal bone microarchitecture [40]. These changes increase bone fragility and fracture susceptibility [41], significantly impacting patients' ability to perform daily activities. In the development of age‐related osteoporosis, cellular senescence is regarded as a key driving factor. The gradual accumulation of senescent cells within the bone microenvironment, particularly osteoblasts and their precursor cells, mediates a local inflammatory state through the continuous release of the senescence‐associated secretory phenotype (SASP) [42], thereby disrupting the normal balance of bone metabolism [43]. In both the oxidative stress‐induced ageing model and the replicative ageing model established in this study, elevated levels of P16 and P21 were observed in both protein and mRNA expression, accompanied by an increase in β‐gal(+) cells, indicating the successful establishment of both ageing models. At the animal level, the number of trabeculae in aged mice significantly decreased with advancing age, accompanied by reductions in trabecular thickness, bone volume fraction, and bone surface area/tissue volume ratio, along with increased trabecular separation. Multiple inflammatory factors in SASP not only suppress the osteogenic function of osteoblasts but also enhance the bone resorption activity of osteoclasts. Simultaneously, they influence the differentiation direction of bone marrow mesenchymal stem cells (BMSCs), making them more prone to differentiate into adipocytes rather than osteoblasts, thereby exacerbating bone loss [44, 45]. Notably, studies indicate that eliminating osteoclast precursors does not effectively mitigate bone loss in age‐related osteoporosis. The fundamental reason lies in the fact that this form of osteoporosis is characterized by the massive accumulation of senescent osteoblast lineage cells within bone tissue, rather than a significant increase in osteoclast activity [42]. Therefore, targeted interventions specifically directed at senescent osteoblasts hold promise as an effective therapeutic strategy for age‐related osteoporosis.

Epigenetics primarily focuses on mechanisms of gene expression regulation that do not involve alterations in DNA sequences [46, 47]. Common mechanisms include DNA and RNA methylation, histone chemical modifications, and the regulatory roles of non‐coding RNAs (ncRNAs) [48, 49, 50]. In the field of osteoporosis research, the epigenetic perspective provides new theoretical insights into understanding its pathogenesis and bone repair processes [51, 52]. Although epigenetic regulation has been recognized as one of the pathways influencing bone homeostasis, the specific mechanisms underlying epigenetic regulation of osteoblast senescence in osteoporosis remain to be further elucidated. Prmt6 is a key member of the PRMT family [53], primarily functioning to catalyse asymmetric dimethylation (aDMA) of arginine residues in both histone and non‐histone substrates [54, 55]. Research indicates that Prmt6 specifically mediates asymmetric dimethylation of histone H3 at position 2 (H3R2), forming the H3R2me2a modification [56]. This modification, acting as an epigenetic mark for transcriptional repression, is mutually exclusive with the active histone modifications H3K4me2 and H3K4me3, playing a crucial role in gene transcription regulation. Compared to advances in osteoclast research, studies on the role of Prmt6 in osteoblast biology, particularly during osteoblast senescence, remain relatively scarce. In this study, Western blot, qPCR, and immunofluorescence results demonstrated that in both ageing models, mRNA and protein expression levels of Prmt6 were significantly reduced in aged osteoblasts, accompanied by a corresponding decrease in β‐gal(+) cell numbers. This indicates that Prmt6 is downregulated in aged osteoblasts. Additionally, Prmt6 knockdown promotes osteoblast senescence, a conclusion supported by immunofluorescence staining results, increased β‐gal(+) cell numbers, and reduced expression of senescence markers. Furthermore, reduced expression of Alp, Ocn, and Runx2 in Prmt6‐knockout osteoblasts suggests that osteogenic differentiation is suppressed or that osteogenic capacity is diminished. This is specifically manifested by impaired Runx2‐driven osteogenic transcriptional activity and a delay in the entire process from early matrix maturation (Alp) to late mineralization (Ocn), further indicating that Prmt6 knockout leads to osteoblast senescence. To further investigate the biological functions of Prmt6, we performed RNA sequencing. The results revealed that osteoclast senescence drives the pathological process of osteoporosis through two core pathways: intrinsic functional decline and chronic inflammation mediated by the secretion of SASP. The imbalance in the ‘inflammation‐vascular‐matrix’ triad and the disruption of the ‘immune‐endocrine‐cell fate’ network can both be regarded as secondary effects and systemic manifestations of osteoblast senescence. At the animal level, Prmt6 and H3R2me2a levels decrease with advancing age in mice. This provides crucial bioinformatics evidence for targeting cellular senescence as a therapeutic approach. This study identified a total of 682 upregulated and 1796 downregulated differentially expressed genes. KEGG enrichment analysis revealed significant enrichment in the ECM‐receptor interaction, PI3K‐Akt, and Rap1 signalling pathways, which have been widely reported to be involved in the regulation of osteoblast proliferation, differentiation, and senescence [57]. Although multiple pathways are associated with bone ageing, this study primarily focuses on the regulation of immune ageing mediated by Prmt6‐mediated epigenetic modifications. Among the enrichment results, the Sting‐related innate immune pathway showed the most significant changes and was highly consistent with the Prmt6 knockout phenotype; therefore, functional validation was prioritized for Sting and its downstream target, Ifitm3. The remaining pathways also hold research value and may be explored further as future directions.

The cGAS‐STING pathway is central to the innate immune system, responsible for sensing cytoplasmic DNA and initiating immune responses [58, 59]. Recent studies have revealed that this pathway also plays a crucial role in cellular senescence [60, 61]. In the skeletal system, abnormal activation of the Sting pathway is closely associated with the development and progression of age‐related osteoporosis [62]. Research indicates that Sting participates in ageing‐related osteoporosis by regulating the HK2‐VDAC1 mitochondrial axis. As the ageing process progresses, ageing‐related factors induce increased Sting expression, thereby disrupting the interaction between hexokinase 2 (HK2) and voltage‐dependent anion channel 1 (VDAC1). Hk2 dissociation from Vadc1 triggers the opening of the mitochondrial permeability transition pore (mPTP), leading to mitochondrial dysfunction and abnormal osteogenic differentiation, thereby disrupting bone homeostasis. These findings establish Sting as an intracellular metabolic checkpoint that promotes osteoporosis development by affecting mitochondrial function [63]. In this study, Western blot, qPCR, and immunofluorescence results demonstrated that in both ageing models, mRNA and protein expression levels of Sting were significantly reduced in aged osteoblasts, while the number of β‐gal(+) cells correspondingly increased, indicating high Sting expression in aged osteoblasts. This study confirms that Sting knockout reduces the expression of ageing markers, and the number of β‐gal(+) cells in Sting ko osteoblasts also decreases accordingly. Furthermore, the expression levels of Alp, Ocn, and Runx2 were elevated in Sting‐knockout osteoblasts, suggesting that Sting knockout inhibits osteoblast senescence, enhances osteogenic differentiation, or increases osteogenic activity. Among these, Runx2 acts as the primary transcription factor that initiates the osteogenic program, while Alp and Ocn reflect accelerated early matrix maturation and late mineralization, respectively. At the animal level, Sting expression in femoral tissue increases with age in mice. This mechanism not only enriches our understanding of the molecular mechanisms underlying bone ageing but also provides a theoretical basis for targeting the Sting pathway to treat age‐related osteoporosis. At the molecular level, Prmt6 forms H3R2me2a modifications, recruiting transcription‐repressive complexes to the promoter regions of target genes, thereby inhibiting gene transcription [28]. It has been demonstrated that Prmt6 functions as a transcriptional repressor for multiple genes, including HOXA2, THBS1, and TP53 [64, 65]. In the context of osteoblast senescence research, we observed a significant reduction in H3R2me2a expression levels in senescent osteoblasts. Concurrently, its expression levels also decreased in Prmt6 ko osteoblasts, confirming that H3R2me2a is catalysed by Prmt6. A similar regulatory mechanism may also exist within the Sting pathway. The absence of Prmt6 disrupts the H3R2me2a modification pattern, triggering genome‐wide chromatin remodelling. This remodelling is particularly pronounced in the regulatory regions of the Sting gene, manifesting as significantly enhanced chromatin accessibility and substantially increased chromatin openness. The open chromatin structure facilitates easier access for transcription factors and other regulatory proteins to the promoter and enhancer regions of the Sting gene, ultimately resulting in abnormal increases in both Sting transcript and protein levels.

Ifitm3 is a small‐molecule transmembrane protein induced by interferon, serving as a key effector molecule in the host's innate immune defence system. Its function was initially recognized in academic circles for its broad‐spectrum antiviral activity, particularly by altering the properties of late endosomal/lysosomal membranes to block the membrane fusion process between various enveloped viruses—such as influenza and dengue viruses—and host cell membranes, thereby preventing viral genomes from entering the cytoplasm [66, 67]. Recent studies have revealed that the function of Ifitm3 extends beyond antiviral activity, as it also participates in regulating various cellular processes including cell adhesion, differentiation, and signal transduction [68]. Furthermore, it is closely implicated in pathological processes such as tumour progression and neurodegenerative diseases [69]. As a downstream effector molecule of the Sting pathway, Ifitm3 is a classic interferon‐stimulated gene (ISG). Upon activation of the Sting pathway, it induces type I interferon production, which in turn significantly upregulates Ifitm3 expression. However, the role of Ifitm3 in osteoblast senescence during osteoporosis remains poorly understood. In the two senescent osteoblast models established in this study and in Prmt6‐deficient osteoblasts, Ifitm3 expression was elevated in the senescent group compared to the control group, whereas Ifitm3 expression was reduced in Sting knockout osteoblasts. This result validates the aforementioned regulatory relationship. The activated Sting signalling pathway further upregulates the expression of its downstream target gene Ifitm3. Ifitm3 expression induces sustained innate immune responses and cell cycle arrest, ultimately driving osteoblasts into a senescent state. Concurrently, reduced numbers of β‐gal(+) cells and decreased expression of senescence markers in Ifitm3 knockout osteoblasts further substantiate Ifitm3's pivotal role in osteoblast senescence. In addition, the expression levels of Alp, Ocn, and Runx2 were significantly elevated in Ifitm3‐knockout osteoblasts, suggesting that Ifitm3 knockout not only inhibits osteoblast senescence but also enhances osteogenic differentiation or increases osteogenic activity. At the animal level, Ifitm3 expression in femoral tissue increases with age in mice. Experimental data from this study indicate that Prmt6 deficiency not only directly upregulates Sting protein and mRNA levels but also enhances its transcriptional activity by increasing chromatin accessibility, ultimately leading to the upregulation of the downstream effector molecule Ifitm3.

Despite these findings, several limitations regarding the molecular mechanism should be addressed here. Notably, our mechanistic exploration has certain limitations. Although Prmt6 deficiency reduces H3R2me2a modification and alters chromatin accessibility at the Sting locus, our current data only demonstrate a correlation instead of direct causality. We cannot confirm that H3R2me2a modification directly mediates the regulatory effect of Prmt6 on Sting chromatin accessibility. Moreover, Prmt6 may modulate Sting chromatin state via other H3R2me2a‐independent pathways. Further rescue experiments will be performed in future studies to verify this regulatory axis. Genetic experiments have established that Ifitm3 lies downstream of the Prmt6/Sting pathway, but the specific effector molecules through which it transmits ageing signals remain unclear. As a transmembrane protein, Ifitm3 regulates processes such as cholesterol binding [70], membrane fluidity, and autophagosome‐lysosome fusion; theoretically, it could promote ageing by interfering with membrane signalling, increasing SA‐β‐Gal activity, or blocking autophagic clearance. However, current data cannot distinguish the relative contributions of these mechanisms in osteoblasts, nor can they determine whether Ifitm3 directly drives senescence or merely acts as a permissive factor for maintaining the lysosomal/autophagic environment. We are currently validating these hypotheses through targeted experiments using cholesterol‐binding mutants, lysosomal pH modulators, and real‐time monitoring of autophagic flux.

## 5 Conclusion

In summary, this study elucidates the pivotal role of the epigenetic regulator Prmt6 in age‐related osteoporosis. Downregulation of Prmt6 expression in senescent osteoblasts disrupts H3R2me2a‐mediated chromatin homeostasis. Its absence specifically enhances accessibility to the Sting locus and activates its expression, thereby upregulating downstream Ifitm3. This drives cellular senescence and bone metabolic imbalance, ultimately promoting osteoporosis development. This discovery establishes the first integrated link between Prmt6‐mediated epigenetic modifications, the Sting innate immune pathway, and cellular senescence, offering novel insights into bone ageing mechanisms. The open state of the Sting locus may serve as a potential biomarker, while targeting the Prmt6/Sting/Ifitm3 axis provides new therapeutic targets for developing intervention strategies against age‐related osteoporosis. However, the precise function of this regulatory axis within the complex in vivo bone microenvironment requires further validation. The long‐term efficacy and safety of epigenetic drugs targeting this pathway warrant systematic evaluation, and whether this mechanism exhibits tissue universality necessitates subsequent in‐depth exploration.

## Author Contributions

**Yuxue Wang:** visualization, methodology, conceptualization, writing – original draft. **Shengjie Chang:** methodology, validation, investigation, supervision, resources. **Ke Xu:** methodology, validation, investigation, supervision, resources. **Xiaoyong Ge:** conceptualization, methodology, visualization, writing – original draft. **Xiaowei Liu:** writing – review and editing, conceptualization. **Haowei Xu:** methodology, validation, investigation, supervision, resources. **Shanjin Wang:** conceptualization, writing – review and editing. **Diankai Wang:** validation, supervision, resources, investigation, methodology.

## Funding

This work was funded by the special Livelihood Research Project for Public Institutions under the Pudong New Area Science and Technology Development Fund (PKJ2024‐Y35), the Key Specialty Construc‐tion Project of Pudong New Area Geriatric Hospital (Grant No. 2025LNYNxk‐A03), the Joint research project of Health Science and Technology project of Shanghai Pudong New Area Health Commission (PW2023D‐12) and the Shanghai East Hospital Specialty Development Project (2024‐DFTS‐009).

## Consent

The authors have nothing to report.

## Conflicts of Interest

The authors declare no conflicts of interest.

## Supporting information


**Table S1:** Primer pairs for real‐time PCR.

## Data Availability

The data sets generated in this study are available on reasonable request from the corresponding author.

## References

[1] Y.‐Y. Zhang , N. Xie , X. D. Sun , et al., “Insights and Implications of Sexual Dimorphism in Osteoporosis,” Bone Research 12 (2024): 8. 10.1038/s41413-023-00306-4 PMC10874461 38368422

[2] L. Ravazzano , G. Colaianni , A. Tarakanova , et al., “Multiscale and Multidisciplinary Analysis of Aging Processes in Bone,” NPJ Aging 10 (2024): 28. 10.1038/s41514-024-00156-2 PMC11180112 38879533

[3] F. Cosman , E. M. Lewiecki , R. Eastell , et al., “Goal‐Directed Osteoporosis Treatment: ASBMR/BHOF Task Force Position Statement 2024,” Journal of Bone and Mineral Research 39 (2024): 1393–1405. 10.1093/jbmr/zjae119 PMC11425703 39073912

[4] K. Wang , “The Potential Therapeutic Role of Curcumin in Osteoporosis Treatment: Based on Multiple Signaling Pathways,” Frontiers in Pharmacology 15 (2024): 1446536. 10.3389/fphar.2024.1446536 PMC11338871 39175539

[5] N. Jianu , V. O. Buda , D. Căpățână , et al., “Osteoporosis: A Problem Still Faulty Addressed by the Romanian Healthcare System. Results of a Questionnaire Survey of People Aged 40 Years and Over,” Frontiers in Medicine 11 (2024): 1485382. 10.3389/fmed.2024.1485382 PMC11537941 39507714

[6] L. Mao , Z. Xia , L. Pan , et al., “Deep Learning for Screening Primary Osteopenia and Osteoporosis Using Spine Radiographs and Patient Clinical Covariates in a Chinese Population,” Frontiers in Endocrinology 13 (2022): 971877. 10.3389/fendo.2022.971877 PMC9513384 36176468

[7] E. Hsieh , D. Bryazka , K. L. Ong , et al., “The Global, Regional, and National Burden Attributable to Low Bone Mineral Density, 1990–2020: An Analysis of a Modifiable Risk Factor From the Global Burden of Disease Study 2021,” Lancet Rheumatology 7 (2025): e873–e894. 10.1016/S2665-9913(25)00105-5 PMC12623303 40972625

[8] J. Zhang , Y. Wang , J. Guo , et al., “The Association Between Ten Anthropometric Measures and Osteoporosis and Osteopenia Among Postmenopausal Women,” Scientific Reports 15 (2025): 10994. 10.1038/s41598-025-94218-4 PMC11958737 40164628

[9] U. C. Ugwu and O. C. Ene , “Status of Osteoporosis in Postmenopausal Women in Rural Communities of Southeast Nigeria: A Cross‐Sectional Study,” BMC Women's Health 25 (2025): 366. 10.1186/s12905-025-03928-4 PMC12291332 40713560

[10] W. Zhao , J. Li , T. Su , et al., “Osteoclast Activation and Inflammatory Bone Diseases: Focusing on Receptors in Osteoclasts,” Journal of Inflammation Research 18 (2025): 3201–3213. 10.2147/JIR.S507269 PMC11890017 40059949

[11] K. Xu , J. Li , R. Wen , B. Chang , Y. Cheng , and X. Yi , “Role of SIRT3 in Bone Homeostasis and Its Application in Preventing and Treating Bone Diseases,” Frontiers in Pharmacology 14 (2023): 1248507. 10.3389/fphar.2023.1248507 PMC10773770 38192409

[12] Q.‐P. Liu and S.‐T. Chai , “Caffeine Intake Is Inversely Associated With Osteoporosis Risk Based on Cross‐Sectional and Genetic Evidence,” Scientific Reports 15 (2025): 20720. 10.1038/s41598-025-07916-4 PMC12216410 40595207

[13] G. Li , Z. Gu , Y. He , C. Wang , and J. Duan , “The Effect of SOX4 Gene 3′UTR Polymorphisms on Osteoporosis,” Journal of Orthopaedic Surgery and Research 16 (2021): 321. 10.1186/s13018-021-02454-x PMC8130251 34006298

[14] K. Jonischkies , M. del Angel , Y. E. Demiray , A. Loaiza Zambrano , and O. Stork , “The NDR Family of Kinases: Essential Regulators of Aging,” Frontiers in Molecular Neuroscience 17 (2024): 1371086. 10.3389/fnmol.2024.1371086 PMC11129689 38803357

[15] U. Föger‐Samwald , K. Kerschan‐Schindl , M. Butylina , and P. Pietschmann , “Age Related Osteoporosis: Targeting Cellular Senescence,” International Journal of Molecular Sciences 23 (2022): 2701. 10.3390/ijms23052701 PMC8910503 35269841

[16] Z. Zhang , P. Liu , Y. Song , et al., “Addressing Osteoblast Senescence: Molecular Pathways and the Frontier of Anti‐Ageing Treatments,” Clinical and Translational Medicine 15 (2025): e70417. 10.1002/ctm2.70417 PMC12274116 40681473

[17] M. V. Wrona , R. Ghosh , K. Coll , C. Chun , and M. J. Yousefzadeh , “The 3 I's of Immunity and Aging: Immunosenescence, Inflammaging, and Immune Resilience,” Frontiers in Aging 5 (2024): 1490302. 10.3389/fragi.2024.1490302 PMC11521913 39478807

[18] M. S. Tahir , J. Karagiannis , and L. Tian , “HD2A and HD2C Co‐Regulate Drought Stress Response by Modulating Stomatal Closure and Root Growth in Arabidopsis,” Frontiers in Plant Science 13 (2022): 1062722. 10.3389/fpls.2022.1062722 PMC9727301 36507458

[19] F. J. Emerson and S. S. Lee , “Chromatin: The Old and Young of It,” Frontiers in Molecular Biosciences 10 (2023): 1270285. 10.3389/fmolb.2023.1270285 PMC10591336 37877123

[20] J. Wu , Y. Liu , Y. Song , L. Wang , J. Ai , and K. Li , “Aging Conundrum: A Perspective for Ovarian Aging,” Frontiers in Endocrinology 13 (2022): 952471. 10.3389/fendo.2022.952471 PMC9437485 36060963

[21] R. Wen , R. Huang , M. Yang , J. Yang , and X. Yi , “Regulation of Protein Arginine Methyltransferase in Osteoporosis: A Narrative Review,” Frontiers in Cell and Developmental Biology 13 (2025): 1453624. 10.3389/fcell.2025.1453624 PMC12058719 40342926

[22] Z. Chen , J. Gan , Z. Wei , et al., “The Emerging Role of PRMT6 in Cancer,” Frontiers in Oncology 12 (2022): 841381. 10.3389/fonc.2022.841381 PMC8931394 35311114

[23] Y. Lei , P. Han , and D. Tian , “Protein Arginine Methyltransferases and Hepatocellular Carcinoma: A Review,” Translational Oncology 14 (2021): 101194. 10.1016/j.tranon.2021.101194 PMC8353347 34365222

[24] F. Inoue , K. Sone , K. Kumegawa , et al., “Inhibition of Protein Arginine Methyltransferase 6 Activates Interferon Signaling and Induces the Apoptosis of Endometrial Cancer Cells via Histone Modification,” International Journal of Oncology 64 (2024): 32. 10.3892/ijo.2024.5620 PMC10836505 38299254

[25] B. Xiong , “Allosteric Modulation: Dynamics Is Double‐“E”dged,” Journal of Medicinal Chemistry 64 (2021): 3694–3696. 10.1021/acs.jmedchem.1c00473 33761250

[26] J. J. Hamey , S. Rakow , C. Bouchard , et al., “Systematic Investigation of PRMT6 Substrate Recognition Reveals Broad Specificity With a Preference for an RG Motif or Basic and Bulky Residues,” FEBS Journal 288 (2021): 5668–5691. 10.1111/febs.15837 33764612

[27] J. W. Hwang , Y. Cho , G.‐U. Bae , S.‐N. Kim , and Y. K. Kim , “Protein Arginine Methyltransferases: Promising Targets for Cancer Therapy,” Experimental & Molecular Medicine 53 (2021): 788–808. 10.1038/s12276-021-00613-y PMC8178397 34006904

[28] X. Zhang , Y. Liu , F. Xu , et al., “Protein Arginine Methyltransferase‐6 Regulates Heterogeneous Nuclear Ribonucleoprotein‐F Expression and Is a Potential Target for the Treatment of Neuropathic Pain,” Neural Regeneration Research 20 (2025): 2682–2696. 10.4103/NRR.NRR-D-23-01539 PMC11801299 39503430

[29] Y. Luo , G. Liang , Q. Zhang , and B. Luo , “The Role of cGAS‐STING Signaling Pathway in Colorectal Cancer Immunotherapy: Mechanism and Progress,” International Immunopharmacology 143 (2024): 113447. 10.1016/j.intimp.2024.113447 39515043

[30] B. M. Freyter , M. A. Abd al‐razaq , A. Isermann , et al., “Nuclear Fragility in Radiation‐Induced Senescence: Blebs and Tubes Visualized by 3D Electron Microscopy,” Cells 11 (2022): 273. 10.3390/cells11020273 PMC8774169 35053389

[31] D. Wang , H. Zhao , Y. Shen , and Q. Chen , “A Variety of Nucleic Acid Species Are Sensed by cGAS, Implications for Its Diverse Functions,” Frontiers in Immunology 13 (2022): 826880. 10.3389/fimmu.2022.826880 PMC8854490 35185917

[32] M. Tian , F. Li , H. Pei , X. Liu , and H. Nie , “The Role of the cGAS‐STING Pathway in Chronic Pulmonary Inflammatory Diseases,” Frontiers in Medicine 11 (2024): 1436091. 10.3389/fmed.2024.1436091 PMC11557406 39540037

[33] S. Zhong , W. Li , Y. Bai , et al., “Computational Study on New Natural Compound Agonists of Stimulator of Interferon Genes (STING),” PLoS One 14 (2019): e0216678. 10.1371/journal.pone.0216678 PMC6532845 31120925

[34] R. Cancado de Faria , L. Silva , B. Teodoro‐Castro , K. S. McCommis , E. V. Shashkova , and S. Gonzalo , “A Non‐Canonical cGAS‐STING Pathway Drives Cellular and Organismal Aging,” *BioRxiv*, (2025), 10.1101/2025.04.03.645994. PMC12280946 40638086

[35] V. Kumar and J. H. Stewart , “cGLRs Join Their Cousins of Pattern Recognition Receptor Family to Regulate Immune Homeostasis,” International Journal of Molecular Sciences 25 (2024): 1828. 10.3390/ijms25031828 PMC10855445 38339107

[36] X. Tang , Y. He , M. Liang , et al., “Neurotoxicity, α‐Synuclein Pathology, and Mitochondrial Dysfunction: A Comparative Study of Different Mouse Models of Parkinson's Disease,” Neural Regeneration Research 21 (2025): 5000–5012. 10.4103/NRR.NRR-D-25-00648 PMC13568611 41017689

[37] C. Yang , Y. Pang , Y. Huang , et al., “Single‐Cell Transcriptomics Identifies Premature Aging Features of TERC‐Deficient Mouse Brain and Bone Marrow,” GeroScience 44 (2022): 2139–2155. 10.1007/s11357-022-00578-4 PMC9616978 35545739

[38] M. F. Gulen , N. Samson , A. Keller , et al., “cGAS–STING Drives Ageing‐Related Inflammation and Neurodegeneration,” Nature 620 (2023): 374–380. 10.1038/s41586-023-06373-1 PMC10412454 37532932

[39] B. D. Paul , S. H. Snyder , and V. A. Bohr , “Signaling by cGAS–STING in Neurodegeneration, Neuroinflammation, and Aging,” Trends in Neurosciences 44 (2021): 83–96. 10.1016/j.tins.2020.10.008 PMC8662531 33187730

[40] Z. Qu , B. Zhang , L. Kong , et al., “Receptor Activator of Nuclear Factor‐κB Ligand‐Mediated Osteoclastogenesis Signaling Pathway and Related Therapeutic Natural Compounds,” Frontiers in Pharmacology 13 (2022): 1043975. 10.3389/fphar.2022.1043975 PMC9683337 36438811

[41] J. Zhang , Z. Sun , L. Xu , Y. Wang , Y. Wang , and B. Dong , “Unraveling the Link Between Metabolic Dysfunction‐Associated Steatotic Liver Disease and Osteoporosis: A Bridging Function of Gut Microbiota,” Frontiers in Endocrinology 16 (2025): 1543003. 10.3389/fendo.2025.1543003 PMC11997446 40235664

[42] J.‐z. Xu , Y. M. Zhou , L. L. Zhang , et al., “BMP9 Reduces Age‐Related Bone Loss in Mice by Inhibiting Osteoblast Senescence Through Smad1‐Stat1‐P21 Axis,” Cell Death Discovery 8 (2022): 254. 10.1038/s41420-022-01048-8 PMC9076651 35523787

[43] L. Fu , P. Zhang , Y. Wang , and X. Liu , “Microbiota‐Bone Axis in Ageing‐Related Bone Diseases,” Frontiers in Endocrinology 15 (2024): 1414350. 10.3389/fendo.2024.1414350 PMC11284018 39076510

[44] H. He , Y. Zhang , Y. Sun , et al., “Folic Acid Attenuates High‐Fat Diet‐Induced Osteoporosis Through the AMPK Signaling Pathway,” Frontiers in Cell and Developmental Biology 9 (2022): 791880. 10.3389/fcell.2021.791880 PMC8762056 35047504

[45] K. Bao , Y. Jiao , L. Xing , F. Zhang , and F. Tian , “The Role of Wnt Signaling in Diabetes‐Induced Osteoporosis,” Diabetology & Metabolic Syndrome 15 (2023): 84. 10.1186/s13098-023-01067-0 PMC10141960 37106471

[46] X. Tang , M. Chen , G. Xu , et al., “Deciphering Epigenetic Regulation in Postmenopausal Osteoporosis: Current and Future Perspectives,” View 6 (2025): 20250108.

[47] C. Wang , W. Wu , B. Yan , et al., “The Causal Relationship Between Epigenetic Aging and Osteoporosis: A Bi‐Directional Mendelian Randomization Study,” Journal of Translational Genetics and Genomics 9 (2025): 90–99.

[48] L. Ren , Y. Yang , W. Li , et al., “Recent Advances in Epigenetic Anticancer Therapeutics and Future Perspectives,” Frontiers in Genetics 13 (2023): 1085391. 10.3389/fgene.2022.1085391 PMC9845602 36685834

[49] Y. Wang , Q. Liu , L. Deng , et al., “The Roles of Epigenetic Regulation in Graft‐Versus‐Host Disease,” Biomedicine & Pharmacotherapy 175 (2024): 116652. 10.1016/j.biopha.2024.116652 38692061

[50] F. Liu , J. Chen , Z. Li , and X. Meng , “Recent Advances in Epigenetics of Age‐Related Kidney Diseases,” Genes 13 (2022): 796. 10.3390/genes13050796 PMC9142069 35627181

[51] I. V. Zhivodernikov , T. V. Kirichenko , Y. V. Markina , A. Y. Postnov , and A. M. Markin , “Molecular and Cellular Mechanisms of Osteoporosis,” International Journal of Molecular Sciences 24 (2023): 15772. 10.3390/ijms242115772 PMC10648156 37958752

[52] X. Liang , W. Shi , X. Zhang , et al., “Causal Association of Epigenetic Aging and Osteoporosis: A Bidirectional Mendelian Randomization Study,” BMC Medical Genomics 16 (2023): 275. 10.1186/s12920-023-01708-3 PMC10623745 37919683

[53] M. Sun , L. Li , Y. Niu , et al., “PRMT6 Promotes Tumorigenicity and Cisplatin Response of Lung Cancer Through Triggering 6PGD/ENO1 Mediated Cell Metabolism,” Acta Pharmaceutica Sinica B 13 (2023): 157–173. 10.1016/j.apsb.2022.05.019 PMC9939295 36815049

[54] W. Chu , W. Peng , Y. Lu , et al., “PRMT6 Epigenetically Drives Metabolic Switch From Fatty Acid Oxidation Toward Glycolysis and Promotes Osteoclast Differentiation During Osteoporosis,” Advanced Science 11 (2024): e2403177. 10.1002/advs.202403177 PMC11516099 39120025

[55] K. Morita , Y. Hatanaka , S. Ihashi , M. Asano , K. Miyamoto , and K. Matsumoto , “Symmetrically Dimethylated Histone H3R2 Promotes Global Transcription During Minor Zygotic Genome Activation in Mouse Pronuclei,” Scientific Reports 11 (2021): 10146. 10.1038/s41598-021-89334-w PMC8115239 33980975

[56] J. Wang , S. Shen , J. You , et al., “PRMT6 Facilitates EZH2 Protein Stability by Inhibiting TRAF6‐Mediated Ubiquitination Degradation to Promote Glioblastoma Cell Invasion and Migration,” Cell Death & Disease 15 (2024): 524. 10.1038/s41419-024-06920-2 PMC11266590 39043634

[57] X. Tang , Y. Pang , P. Zhu , et al., “Cell Apoptosis and Glucocorticoid‐Induced Osteonecrosis,” Med Research 1 (2025): 207–225.

[58] S. Jeon , Y. Jeon , J. Y. Lim , Y. Kim , B. Cha , and W. Kim , “Emerging Regulatory Mechanisms and Functions of Biomolecular Condensates: Implications for Therapeutic Targets,” Signal Transduction and Targeted Therapy 10 (2025): 4. 10.1038/s41392-024-02070-1 PMC11701242 39757214

[59] F. Xue , Y. K. Liu , X. Y. Chen , et al., “Targeting cGAS‐STING: Modulating the Immune Landscape of Hepatic Diseases,” Frontiers in Immunology 16 (2025): 1498323. 10.3389/fimmu.2025.1498323 PMC11911377 40098962

[60] L. Yu and P. Liu , “cGAS/STING Signalling Pathway in Senescence and Oncogenesis,” Seminars in Cancer Biology 106–107 (2024): 87–102. 10.1016/j.semcancer.2024.08.007 PMC11625615 39222763

[61] S. Wang , T. Huo , M. Lu , et al., “Recent Advances in Aging and Immunosenescence: Mechanisms and Therapeutic Strategies,” Cells 14 (2025): 499. 10.3390/cells14070499 PMC11987807 40214453

[62] S. MacLauchlan , P. Kushwaha , A. Tai , et al., “STING‐Dependent Interferon Signatures Restrict Osteoclast Differentiation and Bone Loss in Mice,” Proceedings of the National Academy of Sciences 120 (2023): e2210409120. 10.1073/pnas.2210409120 PMC10104545 37023130

[63] W. Cai , J. Zhao , Y. Chen , et al., “STING Regulates Aging‐Related Osteoporosis by Mediating the Hk2‐Vdac1 Mitochondrial Axis,” Free Radical Biology and Medicine 225 (2024): 1–14. 10.1016/j.freeradbiomed.2024.09.031 39326680

[64] S. Gupta , R. V. Kadumuri , A. K. Singh , S. Chavali , and A. Dhayalan , “Structure, Activity and Function of the Protein Arginine Methyltransferase 6,” Life 11 (2021): 951. 10.3390/life11090951 PMC8470942 34575100

[65] Q. Chen , Q. Hu , Y. Chen , et al., “PRMT6 Methylation of STAT3 Regulates Tumor Metastasis in Breast Cancer,” Cell Death & Disease 14 (2023): 655. 10.1038/s41419-023-06148-6 PMC10562413 37813837

[66] S. Aftab , E. Nelson , M. Hildreth , and X. Wang , “Silencing RNA‐Mediated Knockdown of IFITM3 Enhances Senecavirus A Replication,” Pathogens 13 (2024): 290. 10.3390/pathogens13040290 PMC11054092 38668245

[67] M. V. Diefenbacher , T. J. Baric , D. R. Martinez , R. S. Baric , N. J. Catanzaro , and T. P. Sheahan , “A Nano‐Luciferase Expressing Human Coronavirus OC43 for Countermeasure Development,” Virus Research 339 (2024): 199286. 10.1016/j.virusres.2023.199286 PMC10714359 38016504

[68] Y. Feng , S. Wang , D. Yang , et al., “Inhibition of IFITM3 in Cerebrovascular Endothelium Alleviates Alzheimer's‐Related Phenotypes,” Alzheimer's & Dementia 21 (2025): e14543. 10.1002/alz.14543 PMC11851164 39807629

[69] Q. Xie , L. Wang , X. Liao , et al., “Research Progress Into the Biological Functions of IFITM3,” Viruses 16 (2024): 1543. 10.3390/v16101543 PMC11512382 39459876

[70] M. A. Rohaim , M. Q. al‐Natour , M. A. Abdelsabour , et al., “Transgenic Chicks Expressing Interferon‐Inducible Transmembrane Protein 1 (IFITM1) Restrict Highly Pathogenic H5N1 Influenza Viruses,” International Journal of Molecular Sciences 22 (2021): 8456. 10.3390/ijms22168456 PMC8395118 34445163

