# Supplementary material for: Prmt6 Deficiency Drives Osteoblast Senescence Promoting Age‐Related Osteoporosis via Epigenetic Remodelling of the H3R2me2a/Sting/Ifitm3 Pathway
Source: J Cell Mol Med. 2026 Sep 18;30(18):e71308. doi: 10.1111/jcmm.71308 (PMC13587703; doi:10.1111/jcmm.71308)
Supplement: Supplementary file 1 [file JCMM-30-e71308-s001.docx]

Supplementary Table1 Primer pairs for real-time PCR

| **Primer Name** | Forward primer | Reverse primer |
| --- | --- | --- |
| **Gapdh** | **AGGTCGGTGTGAACGGATTTG** | **GGGGTCGTTGATGGCAACA** |
| **β-actin** | **TGAGCTGCGTTTTACACCCT** | **TTTGGGGGATGTTTGCTCCA** |
| **P16** | **CCCGAACACTTTCGGTCGTA** | **CAGGAGAGCTGCCACTTTGA** |
| **Prmt6** | **CAACGGATACAGCGTGCTTAT** | **CACCGGCATTCTGAGCATCTT** |
| **Sting** | **CCTCACCGCTCCAAATATCTAG** | **CAGTAGTCCAAGTTCGTGCGA** |
| **Ifitm3** | **CCCCCAAACTACGAAAGAATCA** | **ACCATCTTCCGATCCCTAGAC** |
| **E2f3** | **CTTACAGCAGCAGGCAAAGC** | **ACTTCTTGGTGAGCAGACCG** |
| **Hmox1** | **GGAAATCATCCCTTGCCCC** | **TGTTTGAACTTGGTGGGGCT** |
| **Sirt5** | **CCAGTTGTGTTGTAGACGAAAGC** | **TTCCGAAAGTCTGCCATATTTGA** |
| **Acsl4** | **CTTCCTCTTAAGGCCGGGAGC** | **TCTCTTTGCCATAGCGTTTTTAGA** |
| **Igf2bp2** | **GGAGAACGTGGAGCA** | **CGCAGCGGGAAATCAATCTG** |
| **Elf1** | **TCAAGCCCAGGACGAGAAC** | **AAGGCCTAAGCAGCTTCCAG** |
| **Atf4** | **CCTATAAAGGCTTGCGGCCA** | **CACTGCTGCTGGATTTCGTG** |
| **Gpx4** | **GGAGCCCATTCCTGAACCTT** | **GGTGACGATGATGACACACGAAAC** |
| **P21** | **ATCCTGGTGATGTCCGACCTGTT** | **TGGCGTCTCAGTGGCGAAGT** |
| **Alp** | **GGGCCTGCTCTGTTTCTTCA** | **CTGAGATTCGTCCCTCGCTG** |
| **Ocn** | **ACAAGTCCCACACAGCAGCTT** | **GTCAGCAGAGTGAGCAGAAAGAT** |
| **Runx2** | **ATACCCCCTCGCTCTCTGTT** | **ACATAGGTCCCCATCTGCCT** |
